# Pichia pastoris Exhibits High Viability and a Low Maintenance Energy Requirement at Near-Zero Specific Growth Rates

**DOI:** 10.1128/AEM.00638-16

**Published:** 2016-07-15

**Authors:** Corinna Rebnegger, Tim Vos, Alexandra B. Graf, Minoska Valli, Jack T. Pronk, Pascale Daran-Lapujade, Diethard Mattanovich

**Affiliations:** a Department of Biotechnology, BOKU-University of Natural Resources and Life Sciences, Vienna, Austria; b Department of Biotechnology, Delft University of Technology, Delft, The Netherlands; c School of Bioengineering, University of Applied Sciences FH Campus Vienna, Vienna, Austria; d Austrian Centre of Industrial Biotechnology (ACIB GmbH), Vienna, Austria; USDA Forest Products Laboratory

## Abstract

The yeast Pichia pastoris is a widely used host for recombinant protein production. Understanding its physiology at extremely low growth rates is a first step in the direction of decoupling product formation from cellular growth and therefore of biotechnological relevance. Retentostat cultivation is an excellent tool for studying microbes at extremely low specific growth rates but has so far not been implemented for P. pastoris. Retentostat feeding regimes were based on the maintenance energy requirement (*m_S_*) and maximum biomass yield on glucose (*Y_X_*_/*S*_^max^) estimated from steady-state glucose-limited chemostat cultures. Aerobic retentostat cultivation enabled reproducible, smooth transitions from a specific growth rate (μ) of 0.025 h^−1^ to near-zero specific growth rates (μ < 0.001 h^−1^). At these near-zero specific growth rates, viability remained at least 97%. The value of *m_S_* at near-zero growth rates was 3.1 ± 0.1 mg glucose per g biomass and h, which was 3-fold lower than the *m_S_* estimated from faster-growing chemostat cultures. This difference indicated that P. pastoris reduces its maintenance energy requirement at extremely low μ, a phenomenon not previously observed in eukaryotes. Intracellular levels of glycogen and trehalose increased, while μ progressively declined during retentostat cultivation. Transcriptional reprogramming toward zero growth included the upregulation of many transcription factors as well as stress-related genes and the downregulation of cell cycle genes. This study underlines the relevance of comparative analysis of maintenance energy metabolism, which has an important impact on large-scale industrial processes.

**IMPORTANCE** The yeast Pichia pastoris naturally lives on trees and can utilize different carbon sources, among them glucose, glycerol, and methanol. In biotechnology, it is widely used for the production of recombinant proteins. For both the understanding of life in its natural habitat and optimized production processes, a better understanding of cell physiology at an extremely low growth rate would be of extraordinary value. Therefore, we have grown P. pastoris in a retentostat, which allows the cultivation of metabolically active cells even at zero growth. Here we reached doubling times as long as 38 days and found that P. pastoris decreases its maintenance energy demand 3-fold during very slow growth, which enables it to survive with a much lower substrate supply than baker's yeast.

## INTRODUCTION

The methylotrophic yeast Pichia pastoris (syn. Komagataella spp.) has become increasingly popular as a host for the production of recombinant proteins. Commercial products synthesized in P. pastoris include the recently FDA-approved biopharmaceuticals Jetrea (ocriplasmin) and Kalbitor (ecallantide) (1) as well as enzymes employed in research (trypsin and proteinase K) and industry (phospholipase C and phytase) (2). The vast majority of heterologous proteins produced by P. pastoris are secreted, which enables posttranslational modifications and facilitates downstream processing. The genetic toolbox for P. pastoris continues to expand, with glycoengineered strains (3) as a major breakthrough to establish P. pastoris as a preferred host for the future production of glycosylated therapeutic proteins.

Several studies have indicated that the production of secreted recombinant proteins by yeasts is growth rate dependent (4–8). The low specific secretion rate of yeasts, compared to those of mammalian host systems (9), requires industrial production processes to be operated at high cell densities in order for them to be cost-effective. However, at high biomass concentrations, the rate at which respiratory growth can be sustained is restricted by the oxygen and heat transfer capacities of industrial bioreactors (10). The resulting low specific growth rates in industrial fed-batch processes negatively affect biomass yield and protein productivity, thereby limiting product yields and titers (10, 11). On the other hand, excess biomass represents an undesirable by-product, as its formation requires nonproductive substrate consumption. Clearly, breaking the correlation of growth and productivity would be an attractive asset for P. pastoris as a protein production platform. Gaining insights in the physiology of nongrowing, metabolically active P. pastoris at extremely low growth rates may serve as a first step in this direction.

Controlled cultivation of microorganisms can be achieved in a chemostat. However, technical constraints in the addition of fresh medium to the bioreactor limit the range of dilution rates at which a true continuous feed can be realized. Furthermore, zero growth in a chemostat cannot be achieved at all because cells would wash out. Cultivation of microbes in a controlled manner at extremely low specific growth rates or even under zero-growth regimes can, however, be achieved in a retentostat. This continuous-cultivation tool, first proposed by Herbert in 1961 (12), has recently been used to study several industrially relevant organisms, including the yeast Saccharomyces cerevisiae (13). Retentostats are modified chemostats in which biomass is fully retained by channeling the culture effluent through an internal or external filter device. In this continuous culture, the resulting accumulation of biomass is accompanied by a decreasing availability of the energy substrate per cell and unit of time. This decrease inevitably causes progressive decreases of the specific substrate uptake rate and, consequently, of the specific growth rate. Theoretically, prolonged retentostat cultivation culminates in a situation in which the energy derived from the consumed substrate just suffices to sustain cellular maintenance but can no longer support biomass proliferation. Cellular processes related to maintenance, such as the turnover of cellular components, osmoregulation, and defense against reactive oxygen species (14), can therefore be maintained while cell division has ceased. This nongrowing state is fundamentally distinct from starvation in stationary-phase batch cultures, in which the depletion of an essential nutrient causes cellular deterioration and, eventually, cell death (15). Anaerobic retentostat cultivation of S. cerevisiae demonstrated that the maintenance energy requirement of this yeast is independent of the specific growth rate (16). The energy demand for maintenance varies with the organism and, moreover, depends on culture conditions (13, 17). A low maintenance requirement is especially advantageous for the formation of anabolic products at low growth rates, where maintenance metabolism accounts for a high percentage of the consumed substrate (18, 19).

Recent studies showed that P. pastoris reacts to decreasing growth rates and glucose supply by the downregulation of biosynthetic processes such as gene expression and translation and the upregulation of catabolic processes and stress-responsive genes (7, 20). However, to date, no information is available on how P. pastoris adjusts its physiology at near-zero specific growth rates. Growth of the yeast S. cerevisiae at near-zero growth rates has been investigated in aerobic and anaerobic retentostat cultures, in which it remained highly viable, metabolically active, and remarkably stress tolerant (16, 21). Transcriptome analyses on these near-zero-growth-rate cultures of S. cerevisiae pointed toward an extension of growth-rate-dependent expression trends observed at higher specific growth rates. As the growth rate in the retentostat cultures decreased, biosynthetic processes were downregulated, and genes involved in general stress resistance were induced, which was accompanied by an increased resistance to heat stress (21, 22).

The Crabtree-negative (23) yeast P. pastoris exhibits an ∼10-fold-lower maximum sugar uptake rate than that of S. cerevisiae (24, 25), and its maximum specific growth rate on glucose in synthetic medium under comparable conditions is ∼30% lower (26, 27). Therefore, although S. cerevisiae and P. pastoris show similar growth-rate-dependent transcriptional regulation patterns at higher specific growth rates, their physiological and transcriptional adaptations at near-zero specific growth rates may well be different.

The aim of the present study was to investigate the physiology and transcriptional responses of P. pastoris to aerobic, glucose-limited growth. To this end, aerobic retentostat cultures of this yeast were implemented and used to investigate its growth energetics, robustness, and genome-wide transcriptional responses during extremely slow growth. Based on initial experiments, a strain deleted of the flocculation and filamentous growth regulator gene *FLO8* was used to avoid filter clogging.

## MATERIALS AND METHODS

### Strain and culture conditions.

P. pastoris CBS 7435 was obtained from the Centraal Bureau voor Schimmelcultures (The Netherlands). A mutant of this strain in which open reading frame (ORF) PP7435_Chr4-0252 (*FLO8*) was disrupted was constructed with the split-marker cassette method (28), as adapted for P. pastoris (29). Two 1.5-kb regions located ∼200 bp up- and downstream of the translation start site of the ORF were amplified by using primers A_fw and A_bw as well as primers D_fw and D_bw, respectively (Table 1). The resulting fragments, fragments A and B, were used to flank two ∼1-kb overlapping parts of the KanMX marker cassette (30) (primers B_fw, B_bw, C_fw, and C_bw) by fusion PCR, using overhangs on primers A_bw and D_fw that were homologous to the 5′ and 3′ ends of the respective parts B and C of the resistance marker cassette. The two fused fragments AB and CD were simultaneously transformed into electrocompetent (29) P. pastoris cells by electroporation as described previously (31). Successful integration requires three different recombination events, which resulted in the replacement of a 0.4-kb fragment at the 5′ end of PP7435_Chr4-0252 and its promoter by the KanMX cassette. Correct deletion mutants were verified by PCR with primers located outside the split-marker cassette (Det_fw and Det_bw) and gel electrophoresis. A positive single-colony isolate was selected and named CBS 7435 Δ*flo8*. Genomic DNA for the PCR was isolated on Whatman Elute FTA cards (Whatman, United Kingdom). Cultures for cryostocks were grown overnight in 500-ml shake flasks containing either 100 ml YPG (CBS 7435) or YPG-Geneticin (CBS 7435 Δ*flo8*) (10 g yeast extract, 20 g peptone, 12.6 g glycerol, and 500 mg Geneticin per liter) under conditions of vigorous shaking (180 to 200 rpm) at 25°C. After the addition of glycerol (10%, vol/vol), 1-ml aliquots were stored at −80°C. Precultures for shake flask and bioreactor experiments were inoculated from cryostocks and cultivated in YPD and YPD-Geneticin, respectively (10 g yeast extract, 20 g peptone, 20 g glucose, and 500 mg Geneticin per liter) at 25°C and at 180 rpm overnight. For determination of the maximum specific growth rate (μ_max_) of the Δ*flo8* strain and parental strain CBS 7435, 100 ml of buffered synthetic chemically defined medium (31) was inoculated at an optical density at 600 nm (OD_600_) of 0.8, and the mixture was incubated at 25°C and 180 rpm for 8 h.

**TABLE 1:**
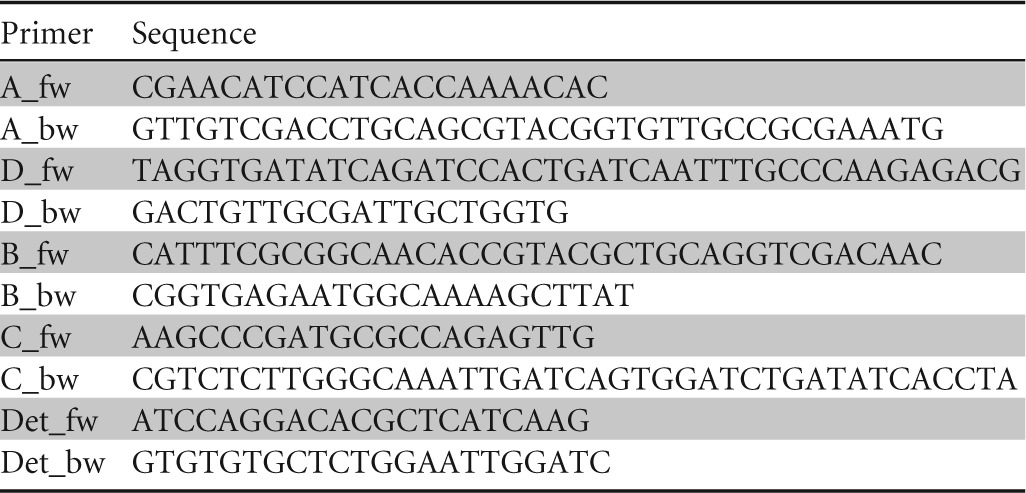
List of primers used for generation of the split-marker cassette

### Chemostat cultivation.

Carbon- and energy-limited aerobic chemostat cultivation for determination of specific glucose uptake rates across four dilution rates (0.025, 0.050, 0.075, and 0.100 h^−1^) and transcriptome analysis were performed with 1.0-liter benchtop bioreactors (SR0700ODLS; Dasgip, Germany). Precultures were grown as described above, harvested, washed and resuspended in sterile demineralized water, and used to inoculate the prefilled bioreactors. After completion of the batch phase (indicated by a sharp increase in the dissolved oxygen concentration), chemostat cultivation was initiated at the respective dilution rate. The working volume of 400 ml was kept steady by means of a level sensor. To keep the dissolved oxygen concentration above 20%, cultures were stirred at 450 rpm and sparged with a constant airflow of 0.2 liters min^−1^. The culture temperature was controlled at 25°C, and the pH was kept at 5.85 by the automated addition of a 10% ammonia solution. Chemostat medium contained (per liter) 10 g glucose, 5 g (NH_4_)_2_SO_4_, 3 g KH_2_PO_4_, 0.5 g MgSO_4_·7H_2_O, 1.5 ml of a trace metals solution (32), 0.4 ml of a 0.2-g liter^−1^ biotin solution (Sigma-Aldrich, USA), and 0.25 g Pluronic 6100 PE antifoaming agent (BASF, Germany).

### Retentostat cultivation.

Retentostat cultivation was preceded by a steady-state chemostat cultivation phase. The retentostat experiment was carried out with 2-liter benchtop bioreactors (Applikon, The Netherlands) with a working volume of 1.4 liters. Main culture conditions (pH and temperature) as well as medium composition were the same as those described above. The sugar concentration in the reservoir medium (*C_S_*_,*MC*_) during the preceding chemostat phase was 10 g liter^−1^. Cultures were sparged with air (0.7 liters min^−1^) and stirred at 800 rpm. Fresh medium was supplied to the bioreactor from a 3-liter mixing reactor (Applikon, The Netherlands), which was operated at a working volume (*V_S_*) of 1.2 liters, and continuously stirred at 500 rpm. *V_S_* was kept constant by means of a level sensor leading to the automatic addition of fresh medium from an external medium vessel at a flow rate (ϕ_*V*_) of 35 ml h^−1^, corresponding to the dilution rate in the bioreactor. Chemostat cultures were considered to be in steady state when, after at least five resident times, biomass concentrations changed by <3% over two consecutive volume changes. After chemostat cultures reached a steady state, the retentostat phase was started by switching the reactor effluent to an outflow port equipped with an autoclavable Applisense filter assembly (Applikon, The Netherlands), consisting of a hydrophobic polypropylene filter with a pore size of 0.22 μm and a stainless steel hollow filter support. Prior to autoclaving, the filter was wetted by overnight incubation in 96% ethanol and subsequently rinsed with a phosphate-buffered saline (PBS) solution (per liter, 8 g NaCl, 0.2 g KCl, 1.44 g Na_2_HPO_4_, 0.24 g KH_2_PO_4_, and HCl to adjust the final pH to 7.4). To control biomass accumulation, the medium reservoir connected to the mixing vessel was exchanged for a reservoir containing chemostat medium (see above) supplemented with 5 g liter^−1^ instead of 10 g liter^−1^ glucose (*C_S_*_,*MR*_). Consequently, the concentration of the growth-limiting substrate glucose entering the bioreactor (*C_S_*_,*in*_) (in grams per liter) decreased over time (*t*) (in hours) according to the following equation:

(1) $$C_{S,in}=\left(C_{S,MC}-C_{S,MR}\right)\cdot e^{\frac{\phi_{V}t}{V_{s}}}+C_{S,MR}$$

In this equation, *C_S_*_,*MC*_ and *C_S_*_,*MR*_ correspond to the glucose concentrations in the medium entering the mixing vessel during the chemostat and retentostat phases, respectively. Sampling frequency and sample volume were minimized to limit the impact of sampling on biomass accumulation inside the reactor. Culture purity was routinely checked by microscopy. Full biomass retention was confirmed by plating the effluent on YPD containing 2% (wt/vol) agar.

### Prediction of retentostat growth kinetics.

To enable a smooth transition of the retentostat cultures to near-zero growth rates, the operational conditions were defined with a mathematical model that simulates growth kinetics of yeast during aerobic retentostat cultivation. Essentially, the mass balance equation for biomass (equation 2) was solved by using the MATLAB ode45 solver by integrating the substrate mass balance (equation 3) with the Herbert-Pirt relation (33) (equation 4):

(2) $$\frac{dC_{X}}{dt}=\mu C_{X}$$

(3) $$\frac{{dC}_{S}}{dt}=\frac{\phi_{V}}{V}(C_{S,in}-C_{S})-q_{S}C_{X}$$

(4) $$q_{S}=\frac{\mu }{Y_{X/S}^{\max\;}}+m_{S}$$

In these equations, *C_X_* (in grams per liter) is the biomass concentration in the retentostat, μ (per hour) is the specific growth rate, *C_S_* (in grams per liter) is the residual substrate concentration, *C_S_*_,*in*_ (in grams per liter) is the substrate concentration in the feed, ϕ_*V*_/*V* (per hour) is the dilution rate, and *q_S_* (in grams substrate per gram of biomass and hour) is the biomass-specific glucose consumption rate. The specific substrate consumption rate can be described by the Herbert-Pirt relation (equation 4), in which *Y_X_*_/*S*_^max^ (in grams per gram) is the maximum biomass yield on glucose and *m_S_* (in grams substrate per gram of biomass and hour) is the maintenance coefficient. Because retentostats were glucose limited and *C_S_*_,*in*_ ≫ *C_S_*, the glucose concentration in the retentostat was assumed to be in a pseudo-steady state such that *dC_S_*/*dt* ≈ 0.

To run simulations, the model required inputs for *m_S_* (assumed to be growth rate independent) and *Y_X/S_*^max^, which were extrapolated from a *q_S_*-μ relationship from duplicate chemostat experiments performed at four different dilution rates between 0.025 h^−1^ and 0.10 h^−1^. In addition, the model required inputs for the variables *V* (in liters), ϕ_*V*_ (in liters per hour), *C_S_*_,*MC*_ (in grams per liter), *C_S_*_,*MR*_ (in grams per liter), and *V_S_* (in liters) and generated time-dependent profiles for biomass accumulation, glucose concentration in the feed, specific glucose consumption rate, and specific growth rate. The final operational conditions chosen for the retentostat experiments are described above. For instructions on how to perform this analysis in MATLAB, including the respective MATLAB codes, see the supplemental material.

### Biomass measurement.

Biomass concentrations (dry weight) were routinely determined in technical duplicates unless stated otherwise. For pre-steady-state chemostat samples, 5 ml of culture broth was harvested, washed with demineralized water, transferred to a preweighed beaker, and dried at 105°C for at least 24 h. Chemostat cultures were considered to be in steady state when, after at least five resident times, biomass concentrations changed by <3% over two consecutive volume changes. Steady-state dry biomass determination was performed by using 25 ml of culture broth. To determine dry mass from the retentostat phase as well as the preceding chemostat phase, samples were kept in demineralized water at an approximate cell concentration of 2.5 mg liter^−1^. Subsequently, exactly 10 ml of the diluted sample was filtered over predried and preweighed membrane filters (pore size, 0.45 μm; Pall Life Sciences, USA), washed with demineralized water, and dried in a microwave oven for 20 min at 350 W. Biomass determination for calculations of μ_max_ from shake flask cultures for the two strains was based on wet cell weight because flocculation disturbed OD_600_ measurements. One milliliter of sample was pipetted in a preweighed Eppendorf tube and centrifuged at 10,000 × *g*. The supernatant was carefully removed, and the tube was weighed again.

### Viability assay.

To measure the viability of retentostat cultures, samples were diluted in PBS to an OD_600_ of 0.1 and double stained with a propidium iodide (PI) stock solution and a 5-carboxyfluorescein diacetate acetoxymethyl ester (5-CFDA,AM) stock solution to final concentrations of 2.0 μmol liter^−1^ and 53.24 μmol liter^−1^, respectively (FungaLight 5-CFDA,AM/PI yeast viability kit; Invitrogen, USA). PI is a DNA-intercalating fluorescence dye that can enter only cells with compromised cell membranes. Binding of PI to DNA leads to a strong increase in red fluorescence. 5-CFDA,AM is a nonpolar and nonfluorescent cell-permeant esterase substrate. Hydrolysis by nonspecific esterases within the cell leads to the generation of polar carboxyfluorescein, a green fluorescent dye, which is no longer cell permeable. Cells were considered viable and metabolically active if they were 5-carboxyfluorescein positive and PI negative. Samples were analyzed on the Accuri flow cytometer (BD Biosciences, USA).

### Cell size measurement.

Bright-field micrographs were converted into 8-bit grayscale images and analyzed with ImageJ. Per reactor and sample point, at least 160 cells were measured by using the elliptical or the freehand selection tool.

### Regression analysis of biomass accumulation in retentostats.

The maintenance energy requirements and biomass-specific death rate of P. pastoris in an aerobic retentostat were estimated from a least-squares regression analysis of data points for the biomass concentration (dry weight) and the viable biomass concentration over time by using a MATLAB model. From these parameters, the specific growth rate and substrate consumption rates were derived. The curve shape was determined by the solution of the following ordinary differential equations with the smallest sum of square errors:

(5) $$\frac{dC_{X\_V}}{dt}=\mu C_{X\_V}-k_{d}C_{X\_V}$$

(6) $$\frac{dC_{X\_d}}{dt}=k_{d}C_{X\_V}$$

(7) $$\frac{dC_{S}}{dt}=\frac{\phi_{V}}{V}(C_{S,in}-C_{S})-q_{S}C_{X\_V}$$

In these equations, *C_X_V_* is the viable-biomass concentration (in grams per liter) and *k_d_* is the death rate (per hour), and equation 4 was used to define the specific substrate uptake rate (*q_S_*). The model required input for the biomass concentrations measured at different time points and the following variables: *V* (in liters), ϕ_*V*_ (in liters per hour), *C_S_*_,*MC*_ (in grams per liter), *C_S_*_,*MR*_ (in grams per liter), *V_S_* (in liters), and *Y_X_*_/*S*_^max^ (in grams per gram). A value for *m_S_* was approximated by using parameter estimation. The time-dependent change of *q_S_* and μ during the course of the retentostat phase followed from the regression analysis. To respect small differences in operational variables per experiment, regression analyses were performed separately on each independent retentostat experiment. For instructions on how to perform this analysis in MATLAB, including the respective MATLAB codes, see the supplemental material and Data Set S1 therein.

### HPLC analysis.

For chemostat cultivations, the glucose concentration in the feed medium was measured with a high-performance liquid chromatography (HPLC) setup (Shimadzu Corporation, Japan) equipped with a Rezex ROA-organic acid H^+^ column (300 mm by 7.8 mm; Phenomenex, USA). A refraction index detector (RID-10A; Shimadzu Corporation, Japan) was used for quantitation. The column was operated at 60°C, the flow rate was set at 1 ml min^−1^, and 4 mM H_2_SO_4_ served as the mobile phase. Before injection, a 900-μl sample was mixed with 100 μl of 0.04 M H_2_SO_4_ and filtered through a 0.20-μm regenerated cellulose (RC) membrane filter (KC90.1; Roth, Germany). The injection volume was 10 μl.

Determinations of the glucose concentration in the mixing vessel, intracellular trehalose contents, and glucose equivalents of intracellular glycogen contents (see below) during retentostat cultivation and the preceding chemostat cultivation were done on an Agilent 1100 HPLC instrument (Agilent Technologies, USA) equipped with an Aminex HPX 87H ion exchange column (Bio-Rad, The Netherlands), operated at 60°C with 5 mM H_2_SO_4_ as the mobile phase at a flow rate of 0.6 ml min^−1^. Detection was performed by means of a dual-wavelength absorbance detector (G1314A; Agilent, USA) and a refractive index detector (G1362A; Agilent, USA).

### Calculations of growth rate dependency of *m_S_* and *Y_X/S_*^max^.

To determine how the maintenance energy substrate requirements and maximum theoretical biomass yield (*m_S_* and *Y_X/S_*^max^, respectively) change with the growth rate, *q_S_* was determined at different μ values in chemostat and retentostat cultures, and regression analysis on moving windows of four duplicate *q_S_* values calculated at four different consecutive μ values was used to determine *m_S_* (intercept at the *y* axis) and *Y_X/S_*^max^ (reciprocal of the slope). Values for μ and *q_S_* from duplicate chemostat cultures were derived by solving the biomass and substrate mass balances under steady-state conditions, respectively. For retentostat cultures, a value of μ between two consecutive sampling points was derived by solving the biomass balance (*dC_X_/dt*) between two time points, *t*_1_ and *t*_2_ (in hours), by using the corresponding biomass concentrations (*C_X_*) (in grams per liter), resulting in

(8) $$\mu =\frac{\ln(\frac{C_{X_{2}}}{C_{X_{1}}})}{t_{2}-t_{1}}$$

The *q_S_* value depends on the amount of the limiting substrate glucose (*S*) (in grams) consumed in the retentostat between certain time points, which follows from the integration of equation 1 multiplied by the constant flow rate at which medium is supplied to the bioreactor (ϕ_*V*_) (in liters per hour), according to

(9) $$S_{2}-S_{1}=(\int_{t_{1}}^{t_{2}}C_{S,in}\;(t))\times \phi_{V}$$

The apparent biomass yield on glucose (*Y_X/S_*) (in grams per gram) in this period can then be calculated according to

(10) $$Y_{X/S}=\frac{(C_{X_{2}}-C_{X_{1}})\;\times \;V}{S_{2}-S_{1}}$$

In this equation, *V* (in liters) represents the bioreactor volume, and the glucose entering the bioreactor is assumed to be completely consumed. Accordingly, *q_S_* can be calculated as

(11) $$q_{S}=\frac{\mu }{Y_{X/S}}$$

Individual *q_S_*-μ plots and corresponding linear regression analyses are shown in Fig. S1 in the supplemental material. Values for *m_S_* and *Y_X/S_*^max^ were derived only from *q_S_*-μ relationships that were considered linear (*R*^2^ > 0.99).

### Analysis of intracellular glycogen and trehalose contents.

Immediately after sampling, 1 ml of broth was added to 5 ml of precooled methanol (−40°C) and mixed, and the mixture was centrifuged at 4,400 × *g* at −19°C for 5 min. Cell pellets were washed with 5 ml precooled methanol and stored at −80°C. For analysis, the pellets were then resuspended and diluted in Na_2_CO_3_ (0.25 M) and further processed as described previously (34). The trehalose levels were measured directly by HPLC, whereas the amount of glucose released from glycogen was measured by HPLC after overnight incubation of the samples with α-amyloglucosidase (from Aspergillus niger; Sigma-Aldrich, The Netherlands) at 57°C.

### RNA extraction, microarray hybridization, and transcriptome data analysis.

Chemostat and retentostat samples for RNA isolation were immediately added in a 2:1 ratio to a precooled fixing solution (5% [vol/vol] phenol in ethanol [absolute]) and centrifuged at 10,000 × *g* for 1 min. Pellets were stored at −80°C before further processing. RNA isolation was done by using Tri reagent according to the supplier's instructions (Ambion, USA). RNA integrity and concentrations were analyzed by using RNA nanochips (Agilent, USA) and a Nanodrop instrument (Thermo Scientific, USA), respectively. For transcriptome analysis, in-house-designed P. pastoris-specific oligonucleotide arrays (AMAD-ID 034821, 8 × 15K custom arrays; Agilent, USA) were used (35). Synthesis of cRNA, hybridization, as well as scanning were carried out according to the Agilent protocol for 2-color expression arrays. Samples were labeled with Cy3 and Cy5 in triplicates and hybridized against a reference pool generated from cells grown under various culture conditions. For all samples, dye swap experiments were carried out.

Normalization steps and statistical analysis of microarray data included background correction with the “normexp” method with an offset of 50 (36) and removal of color bias using locally weighted MA-scatterplot smoothing (LOESS) followed by between-array normalization using the “Aquantile” method. The *P* values associated with the differential expression values were calculated by using a linear model fit and an eBayes adjustment (limma R package). Subsequently, they were adjusted for multiple testing by using the method of Benjamini and Hochberg (BH) (37), using the BH method of the limma R package. For identifying differentially expressed genes, the following criteria were applied: a fold change (FC) cutoff of at least 2 > FC > 1/2 and an adjusted *P* value of <0.05. All steps were done by using the R software package (http://www.rproject.org/) and the limma package. Samples taken from the different bioreactor systems were not directly compared to each other, but log_2_ FC values were calculated in both cases against the chemostat set point of 0.1 h^−1^.

### Cluster analysis and Gene Ontology term enrichment.

Genes that were differentially expressed in at least one comparison (see above) were grouped according to their expression profiles by the Genesis software tool (38), employing *k*-means clustering and Pearson correlation. According to figure-of-merit analysis, a *k* value of 2 was determined to be the ideal number of clusters for the data set. For determination of enriched Gene Ontology (GO) terms in the respective clusters, the online Generic GO Term Finder tool (http://go.princeton.edu/cgi-bin/GOTermFinder) and Saccharomyces Genome Database (SGD) annotations were used. The cutoff for the corrected *P* value (Bonferroni correction) was set to 0.05, and a P. pastoris-specific background list comprised of all annotated genes and genes with unknown function was provided.

### Determination of residual glucose concentrations.

Rapid sampling and quenching of culture broth with cold steel beads were done as described previously (39). Residual glucose concentrations were measured by using the Megazyme d-glucose (glucose oxidase/peroxidase [GOPOD]) assay (Megazymes, Ireland). The protocol provided by the manufacturer was adapted to a 96-well format. Briefly, 60 μl of the sample was mixed with an equal volume of GOPOD reagent. After incubation at 37°C for 30 min, the absorbance at 510 nm was measured with a Tecan plate reader. A standard curve and linear regression were used to calculate residual glucose concentrations. Absorbance-to-glucose-concentration ratios were linear down to 1 mg liter^−1^ glucose.

### Cell cycle distribution analysis.

For fixation, 1 ml of 70% ethanol was added dropwise to 20 μl of the cell culture. This mixture was subsequently stored at −20°C. To measure cell cycle distribution, an aliquot of 350 μl was centrifuged for 3 min at 10,000 × *g* and washed by using PBS-Tween 20 (0.1%), employing the same centrifuge settings. Pellets were resuspended in 1 ml PBS, and 10 μl of a solution containing 100 mg ml^−1^ RNase A (from bovine pancreas; Sigma-Aldrich, USA) was added. After incubation for 1 h at 37°C, samples were pelleted again and washed as described above. Finally, the cell pellets were resuspended in 1 ml PBS and sonicated twice at 85% for 10 s each (UIS250L ultrasonic processor and LTS24d10.4L2 sonotrode; Hielscher, Germany). A 150-μl aliquot of the sonicated cell suspension was mixed with 150 μl of a PI stock solution (4.30 μM) and analyzed on the Gallios flow cytometer (Beckman Coulter, USA).

### Accession number(s).

Transcriptomics data have been deposited at the Gene Expression Omnibus with the accession number GSE81070.

## RESULTS

### Model strain for aerobic retentostat cultivation of P. pastoris.

In recent retentostat studies with S. cerevisiae, biomass retention was achieved by redirecting the effluent of steady-state chemostats through a membrane filter probe (16, 21, 40). When the same setup was used to grow a recombinant P. pastoris strain that secreted human serum albumin (HSA), the filter probe clogged within a few hours after switching to the retentostat mode (data not shown). Since protein aggregation and adsorption are known to cause membrane filter fouling and blockage (41, 42), secreted HSA might have contributed to this problem. However, the total protein concentration in the culture supernatants was only about 115 mg liter^−1^, 20 to 30 mg liter^−1^ of which was HSA, and clogging occurred soon after switching to the retentostat mode. Therefore, we regarded filter clogging by extracellular protein to be improbable. Another possible cause was attachment of P. pastoris to surfaces, a feature previously observed in bioreactor cultures of this yeast (our unpublished observations). Growth on or into the filter probe, which was already present in the bioreactor during the chemostat phase that preceded retentostat cultivation, poses a major risk for its functionality. To eliminate both potential causes of membrane filter obstruction, we set out to construct a nonproducing P. pastoris strain with reduced growth on surfaces.

**FIG 1:**
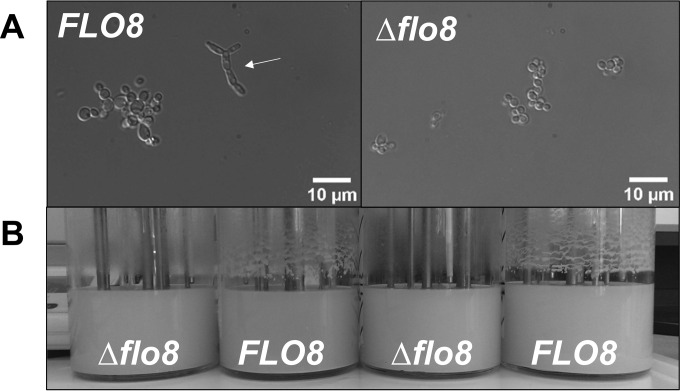
Morphology and surface attachment of wild-type P. pastoris CBS 7435 (*FLO8*) and an isogenic Δ*flo8* strain after 7 days of cultivation in glucose-limited, aerobic chemostat cultures (dilution rate [*D*] = 0.05 h^−1^). (A) Differential interference contrast micrographs. (B) Growth on bioreactor surfaces.

Laboratory strains of S. cerevisiae have been selected for unicellular, noninvasive growth, without flocculation or attachment to surfaces. The loss of these traits has been ascribed to a single nonsense mutation in *FLO8* (43). The transcription factor Flo8 plays an important role in the regulation of flocculation (44, 45) and is part of the filamentous growth signaling pathway (46). Only one putative P. pastoris homolog of S. cerevisiae Flo8 was found by BLAST search. The ORF encoding this protein, PP7435_Chr4-0252, tentatively called *FLO8*, was deleted in P. pastoris CBS 7435 with the split-marker cassette method (28, 29). The impact of the Δ*flo8* mutation was investigated by growing the mutant strain and its *FLO8* parent in glucose-limited chemostat cultures at a dilution rate of 0.05 h^−1^ for 8 residence times (∼1 week). In cultures of the *FLO8* wild-type strain, a considerable fraction of the population showed a pseudohyphal morphology, while Δ*flo8* cultures retained an ovoid, budding morphology (Fig. 1A). Furthermore, attachment of biomass on reactor walls and other reactor components was much less pronounced in the Δ*flo8* strain than in the parental strain (Fig. 1B). Deletion of *FLO8* resulted in a 4% decrease in the biomass yield on glucose in chemostat cultures (*D* = 0.05 h^−1^) (see Table S1 in the supplemental material) and a 12% decrease in the specific growth rate in duplicate shake flask batch cultures (see Table S1 in the supplemental material). These minor effects might be caused by the expression of the dominant selection marker gene KanMX (47). Because the pseudohyphal and invasive phenotype prevented retentostat operation, the Δ*flo8* strain was used for further chemostat and retentostat studies.

### Maintenance energy requirement in glucose-limited chemostat cultures.

When microbial growth is limited by the availability of the energy substrate and specific growth rates are low, a large fraction of energy substrate consumption is invested in cellular maintenance (33). Maintenance includes essential aspects of cellular metabolism that are not coupled to growth, such as macromolecule turnover and homeostasis of electrochemical potential gradients across membranes (14, 19, 48). Biomass accumulation profiles in retentostats strongly depend on the specific maintenance energy requirement (16) under the given experimental conditions. Estimation of the specific rate of energy substrate consumption required for maintenance (*m_S_* [in grams of substrate per gram of biomass and hour]) is therefore a prerequisite for the design of feed profiles for retentostat experiments.

During growth under energy-substrate-limited conditions, microorganisms in which the maintenance energy requirement is growth rate independent show a linear relationship between the specific carbon and energy consumption rate (*q_S_*) and the specific growth rate (μ) according to Pirt (33) (equation 4).

**FIG 2:**
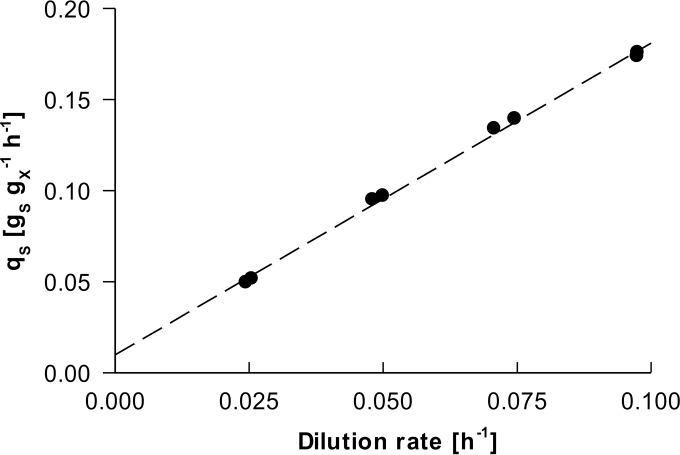
Specific glucose uptake rate (*q_S_*) in aerobic, steady-state, glucose-limited chemostat cultures of P. pastoris grown at different dilution rates. Linear regression was used to calculate the maintenance coefficient, *m_S_* (intercept with the *y* axis), and maximum biomass yield on glucose, *Y_X/S_*^max^ (reciprocal of the slope). The *R*^2^ value of the regression line was 0.99, and the standard error of the maintenance coefficient was determined by the LINEST function to be 23%.

In this equation, *m_S_* represents the growth-rate-independent energy substrate requirement for maintenance, and *Y_X/S_*^max^ (in grams per gram) is the maximum theoretical biomass yield on glucose under the conditions applied. When this equation holds, both parameters can be estimated from a set of steady-state glucose-limited chemostat cultures (in which μ equals the dilution rate) operated at different dilution rates, followed by linear regression of a *q_S_*-versus-μ plot. To calculate *m_S_* and *Y_X/S_*^max^ for the P. pastoris Δ*flo8* strain, eight independent glucose-limited aerobic chemostats were performed, at dilution rates ranging from 0.025 to 0.10 h^−1^. A linear relationship between *q_S_* and μ was indeed observed (Fig. 2), yielding an estimated *m_S_* (± standard deviation) of 0.0100 ± 0.0023 g_*S*_ g_*X*_^−1^ h^−1^. This estimate of *m_S_* was similar to that calculated for S. cerevisiae grown in aerobic retentostat cultures (average ± standard deviation of 0.0071 ± 0.0005 g_*S*_ g_*X*_^−1^ h^−1^) (21). The *m_S_* of the nonproducing P. pastoris strain used in this study was ∼50% lower than the *m_S_* values calculated for heterologous-protein-producing P. pastoris strains grown in glucose-limited chemostats, which ranged from 0.0147 ± 0.0009 g_*S*_ g_*X*_^−1^ h^−1^ (calculated from data in reference 7) to 0.0161 g_*S*_ g_*X*_^−1^ h^−1^ (8). *Y_X/S_*^max^ was calculated to be 0.584 g g^−1^, which is ca. 4% higher than *Y_X/S_*^max^ values of heterologous-protein-producing P. pastoris strains reported previously (0.559 and 0.562 ± 0.011 g g^−1^ [see references 8 and 7, respectively]).

### Higher-than-expected biomass accumulation in retentostats.

**FIG 3:**
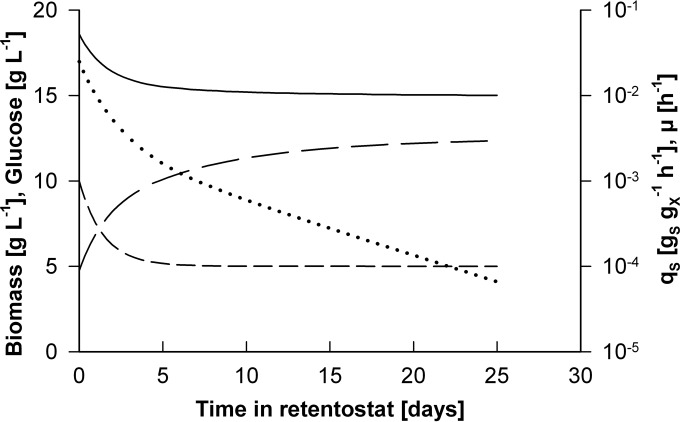
Model-based prediction of growth of P. pastoris in glucose-limited aerobic retentostat cultures. An optimized feed profile, designed to achieve a smooth decline of the specific growth rate (μ) from 0.025 h^−1^ to near-zero growth rates, was designed by computer simulation (see Materials and Methods). Indicated are the optimized glucose concentration profile in the feed (short dashed line), the resulting predicted profiles of biomass accumulation (long dashed line), the specific glucose uptake rate (*q_S_*) (solid line), and μ (dotted line).

A retentostat feeding regime for aerobic, glucose-limited chemostat cultures of the P. pastoris Δ*flo8* strain was designed according to an approach developed previously for aerobic retentostat cultivation of S. cerevisiae (21). Model simulations were based on the *m_S_* and *Y_X/S_*^max^ values for the P. pastoris Δ*flo8* strain estimated from chemostat cultures (see above). Dynamic, declining glucose-feeding profiles were generated by the controlled dilution of the medium feed, using two medium reservoirs that contained media with different glucose concentrations and a mixing vessel upstream of the reactor (see Materials and Methods). Glucose concentrations in the two medium reservoirs (*C_S_*_,*MC*_ and *C_S_*_,*MR*_) and the volume of the mixing vessel were chosen such that the model-based simulations predicted a smooth transition from chemostat growth at 0.025 h^−1^ to near-zero specific growth rates in the retentostats. The optimized feeding strategy and the resulting predicted profiles of biomass accumulation, μ and *q_S_*, are shown in Fig. 3.

**FIG 4:**
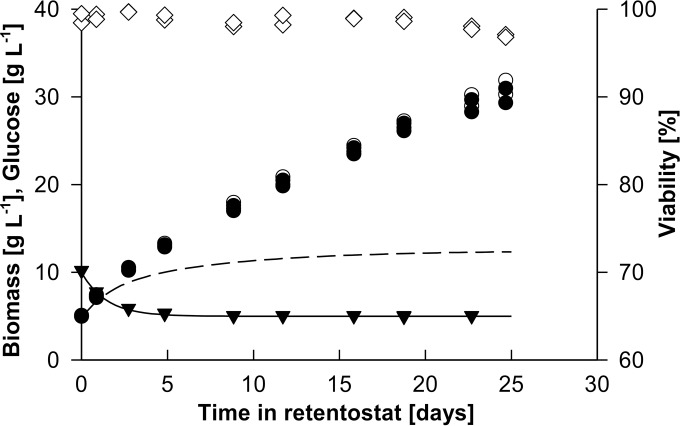
Biomass accumulation, glucose concentration in the feed, and viability of two independent glucose-limited aerobic retentostat cultures of P. pastoris. Retentostat cultures were initiated from chemostat cultures at time zero. Shown are the predicted biomass accumulation profile (short dashed line), measured dry biomass concentration (open circles), biomass concentration corrected for viability (closed circles), predicted glucose concentration (solid line), measured glucose concentration in the mixing vessel (closed triangles), and culture viability based on fluorescence staining (open diamonds).

The optimized feed regime was applied in two independent 25-day retentostat experiments with the P. pastoris Δ*flo8* strain. Although glucose concentrations in the feed exactly matched the predicted profile, biomass accumulated to ∼6-fold-higher concentrations than those predicted by model simulations (Fig. 4). In retentostat cultures of S. cerevisiae, deviation of biomass accumulation from predicted values was attributed to the accumulation of metabolically inactive (nonviable) cells (16, 21). Since the model simulations assumed 100% culture viability, they did not capture possible accumulation of nonviable biomass. Culture viability of the P. pastoris retentostat cultures was regularly measured by fluorescence staining and flow cytometry, which yielded consistent results with plate counts of CFU (see Table S2 in the supplemental material). Throughout the retentostat experiments, the fraction of viable cells remained at 97% or higher (Fig. 4), thereby excluding a loss of viability as a primary cause of the higher-than-predicted level of biomass accumulation. An alternative explanation for this deviation from the model predictions was that the values of *m_S_* and/or *Y_X/S_*^max^ calculated from the chemostat cultures are not representative of those at near-zero growth rates. More precisely, a lower *m_S_* and/or a higher *Y_X/S_*^max^ at near-zero growth rates might contribute to the unexpectedly high level of biomass accumulation. Cell morphology remained unchanged throughout the retentostat culture (see Fig. S2 in the supplemental material). The average cell size increased 1.2-fold from a 3.3- to a 4.2-μm^2^ cross-sectional area, indicating that the cell number increased nearly proportionally to the biomass concentration.

### P. pastoris decreases its maintenance energy requirements at near-zero growth rates.

**FIG 5:**
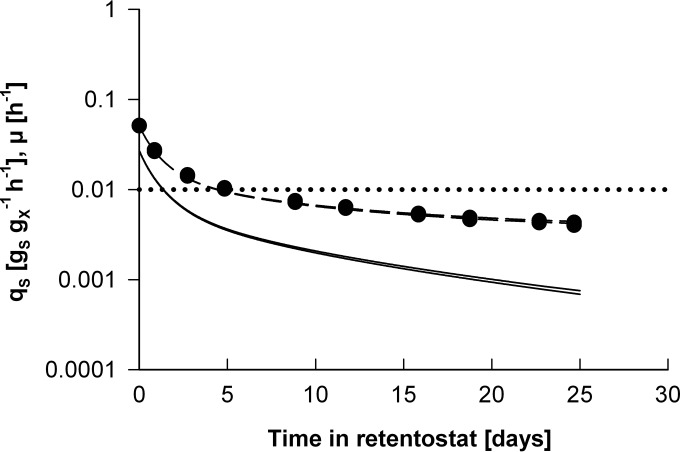
Glucose uptake rate (*q_S_*) and specific growth rate (μ) in two independent glucose-limited aerobic retentostat cultures of P. pastoris. Shown are directly calculated *q_S_* values (closed circles) and values for *q_S_* derived from nonlinear regression analysis of biomass accumulation (short dashed lines) as well as μ derived from nonlinear regression analysis of biomass accumulation (solid lines). The horizontal dotted line indicates the amount of substrate required to meet the maintenance requirement, as extrapolated from chemostat cultivations at higher growth rates.

During the 25-day aerobic retentostat experiments, the specific growth rate of P. pastoris retentostat cultures, calculated by least-squares regression analysis (see Materials and Methods; see also Fig. S3 in the supplemental material), smoothly decreased from 0.025 h^−1^ to below 0.001 h^−1^ (Fig. 5). This decrease in the specific growth rate corresponded to an increase of the doubling time from 27.7 h to over 38 days. Over the same time period, the specific glucose uptake rate declined to 0.004 g_*S*_ g_*X*_^−1^ h^−1^. This extremely low *q_S_* value, which reflects the combined consumption of glucose for maintenance and growth, was only 40% of the *m_S_* value calculated from chemostat cultures grown at specific growth rates of 0.025 h^−1^ and higher. Indeed, the average *m_S_* value calculated by least-squares regression analysis of data from the retentostat cultures equaled 0.0031 ± 0.0001 g_*S*_ g_*X*_^−1^ h^−1^, which is 3-fold lower than the *m_S_* value calculated from the chemostat experiments. This result suggested that in P. pastoris, *m_S_* is not growth rate independent but decreases strongly at extremely low specific growth rates in a way that resembles growth energetics of prokaryotes that exhibit a stringent response (49–51).

**FIG 6:**
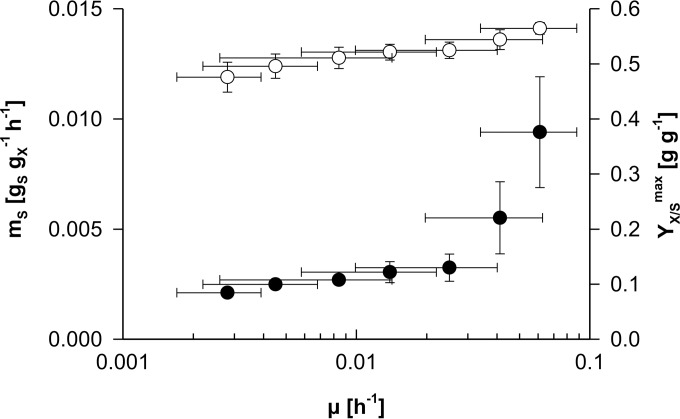
Dynamics of the glucose requirements for maintenance (*m_S_*) and maximum theoretical biomass yield (*Y_X/S_*^max^) in relation to the specific growth rate (μ). Values for *m_S_* (closed circles) and *Y_X/S_*^max^ (open circles) over defined ranges of four consecutive specific growth rates were derived from linear regression analysis performed on overlapping sets of duplicate μ-*q_S_* relations (see Fig. S1 in the supplemental material). The vertical error bars for *m_S_* and *Y_X/S_*^max^ indicate the standard errors of the regression statistics, and the horizontal error bars indicate the defined ranges of specific growth rates.

In addition to changes in *m_S_*, a growth rate dependency of *Y_X/S_*^max^ might affect biomass accumulation profiles in the retentostats. *Y_X/S_*^max^ is among others determined by biomass composition, which can change depending on the environment (52). To investigate this possibility, *q_S_*-μ relationships were determined over a range of specific growth rates in the retentostat and chemostat cultures described in this study. Chemostat data were derived from simple steady-state kinetics, while μ and *q_S_* in the retentostat were calculated by solving biomass and substrate mass balances over defined time intervals (see Materials and Methods). Linear regression analysis was then performed on sets of overlapping μ-*q_S_* relations to estimate *m_S_* and *Y_X/S_*^max^ values over defined ranges of four consecutive specific growth rates in duplicate (see Fig. S1 in the supplemental material). This analysis confirmed that *m_S_* sharply decreased at specific growth rates of between 0.06 h^−1^ and 0.03 h^−1^ (ca. 3-fold) while also indicating a positive but far-less-pronounced correlation of *Y_X/S_*^max^ with the specific growth rate (Fig. 6).

### Glycogen and trehalose accumulation in retentostat-grown P. pastoris cultures.

**FIG 7:**
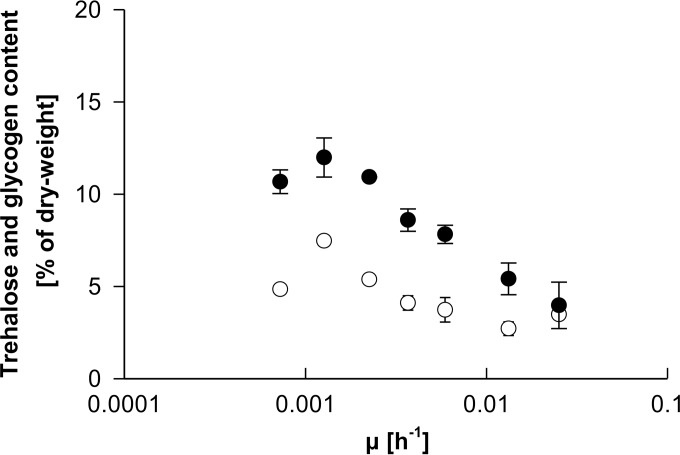
Storage carbohydrate accumulation in aerobic glucose-limited retentostat cultures of P. pastoris. Trehalose (closed circles) and glycogen (open circles) contents are represented as weight percentages of the total dry biomass, plotted as a function of the specific growth rate (μ).

In glucose-limited cultures of S. cerevisiae, intracellular storage carbohydrate concentrations are negatively correlated with the specific growth rate (53). To investigate whether P. pastoris showed a similar response, the glycogen and trehalose contents in biomass were determined during the course of retentostat cultivations (Fig. 7). For all specific growth rates, trehalose levels were higher than glycogen concentrations. Intracellular levels of both storage carbohydrates increased as μ decreased from 0.025 h^−1^ to 0.0013 h^−1^, reaching maximum levels of 0.12 g g_*X*_^−1^ and 0.07 g g_*X*_^−1^, respectively. Average trehalose and glycogen levels measured at the final sampling points, where μ had decreased to 0.0007 h^−1^, were 11 and 35% lower than these maximum levels, respectively. To calculate the fraction of the substrate that cells spent on storage carbohydrate formation, the amount of glucose consumed was related to the amounts of glycogen and trehalose produced in a given time period. At the beginning of retentostat cultivation, ∼6% of glucose was spent on storage carbohydrate formation. This fraction increased to ∼13% when the specific growth rate decreased from 0.0023 to 0.0013 h^−1^ and dropped again to ∼9% at the end of retentostat cultivation.

### Transcriptional reprogramming at near-zero growth rates.

**FIG 8:**
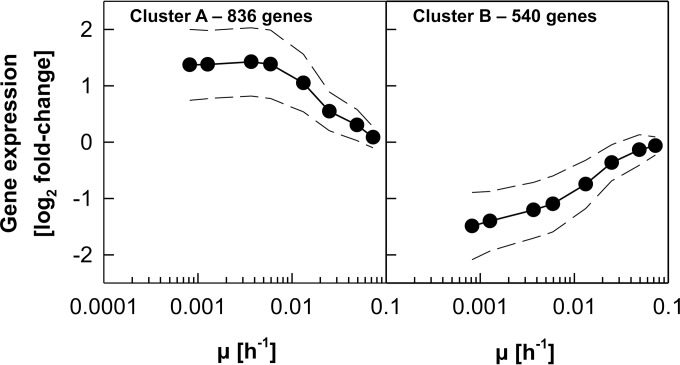
Global gene expression profile in P. pastoris over a wide range of specific growth rates (μ). Genes that were differentially expressed compared to the highest μ of 0.10 h^−1^ in at least one comparison were grouped into two clusters by *k*-means clustering.

Retentostat cultivation enabled a first exploration of the transcriptional adaptations of P. pastoris to near-zero specific growth rates. Microarray-based transcriptome analyses were performed on samples taken on days 1, 3, 5, 16, and 23 of retentostat cultivation, corresponding to average specific growth rates of 0.0132, 0.0059, 0.0037, 0.0013, and 0.00082 h^−1^, respectively. To expand the range of investigated growth rates, transcriptional analysis was also performed on the set of chemostat cultures that were run to estimate *m_S_* and *Y_X/S_*^max^, and these data were combined with the retentostat transcriptome data. Out of 5,354 ORFs represented on the microarray, 1,376 genes were differentially expressed (see Materials and Methods; see also Data Set S2 in the supplemental material). Two main regulation patterns were identified (Fig. 8). The expression of genes either was negatively correlated to the specific growth rate (cluster A; 61% of regulated genes) or displayed the opposite trend, with higher expression levels at higher growth rates (cluster B; 39% of regulated genes).

The gene set and the respective biological processes that were transcriptionally upregulated toward zero growth identified in this study correlate well with regulation patterns previously observed for P. pastoris cultivated in glucose-limited chemostats at μ values ranging from 0.15 to 0.015 h^−1^ (7). Affected biological processes in this study (Table 2) were “cellular response to chemical stimulus,” “cell communication,” and “response to nitrogen utilization.” Furthermore, genes with molecular functions related to aldehyde dehydrogenase activity as well as to promoter binding and transcription factor activity were significantly enriched among the genes with increasing transcript levels toward zero growth (Table 2). In fact, 20% of the genes that were grouped into cluster A are functionally annotated as transcription factors, while in total, only 10% of all P. pastoris ORFs are assigned to this function. Many of the transcription factors located in cluster A are global stress response regulators (*YAP1*, *SKN7*, *SKO1*, *HAC1*, *HSF1*, and *MSN4*), while others are involved in nutrient responses such as glucose repression (*CAT8-2*, *MIG1-1*, and *MIG1-2*) and nitrogen catabolite repression (NCR) (*MKS1*, *URE2-2*, *GAT1*, and *GLN3*). Upregulation of NCR-related transcription factors toward zero growth might seem unexpected, as ammonia was present in excess during chemostat and retentostat cultivations. However, for S. cerevisiae, it has been demonstrated that not only nitrogen but also carbon signaling is responsible for the regulation of NCR (54), which might also be the case for P. pastoris.

**TABLE 2:** Enriched GO terms for the categories “biological process” and “molecular function” for clusters A and B^*a*^

Cluster	Enriched GO term (category)	Corrected *P* value^*b*^
A (genes upregulated toward zero growth)	RNA polymerase II regulatory region sequence-specific DNA binding (MF)	0.00
	Cellular response to chemical stimulus (BP)	0.00
	Sequence-specific DNA binding (MF)	0.00
	Nucleic acid binding transcription factor activity (MF)	0.00
	Cell communication (BP)	0.00
	Regulatory region nucleic acid binding (MF)	0.00
	Response to chemical (BP)	0.00
	Regulatory region DNA binding (MF)	0.00
	RNA polymerase II core promoter proximal region sequence-specific DNA binding transcription factor activity (MF)	0.01
	Aldehyde dehydrogenase (NAD) activity (MF)	0.01
	Oxidoreductase activity, acting on the aldehyde or oxo group of donors (MF)	0.01
	Regulation of nitrogen utilization (BP)	0.01
	Oxidoreductase activity, acting on the aldehyde or oxo group of donors, with NAD or NADP as an acceptor (MF)	0.04
B (genes downregulated toward zero growth)	Mitotic nuclear division (BP)	0.00
	Organelle fission (BP)	0.00
	Chromosome segregation (BP)	0.00
	Cell cycle (BP)	0.00
	Cell division (BP)	0.00
	Microtubule-based process (BP)	0.00
	Cytoskeleton organization (BP)	0.00
	Structural constituent of cytoskeleton (MF)	0.00
	Tubulin binding (MF)	0.00
	Chromosome organization (BP)	0.00
	DNA-dependent DNA replication (BP)	0.00
	DNA conformation change (BP)	0.00
	DNA replication (BP)	0.00
	Single-organism cellular process (BP)	0.00
	Single-organism reproductive process (BP)	0.00
	Single-organism process (BP)	0.00
	Cytoskeletal protein binding (MF)	0.00
	DNA packaging (BP)	0.00
	Nuclear migration (BP)	0.00
	Double-strand break repair (BP)	0.00
	Substrate-specific transmembrane transporter activity (MF)	0.01
	Organic acid transmembrane transporter activity (MF)	0.01
	DNA metabolic process (BP)	0.01
	Small-molecule metabolic process (BP)	0.01
	Sterol metabolic process (BP)	0.02
	Reproduction (BP)	0.02
	Reproductive process (BP)	0.03

a Redundant GO terms were excluded by using the Web-based tool REVIGO (73). For the full-length list as well as the number of significantly regulated genes per GO term and GO term size, see Data Set S2 in the supplemental material. BP, biological process; MF, molecular function.

b Bonferroni correction.

**FIG 9:**
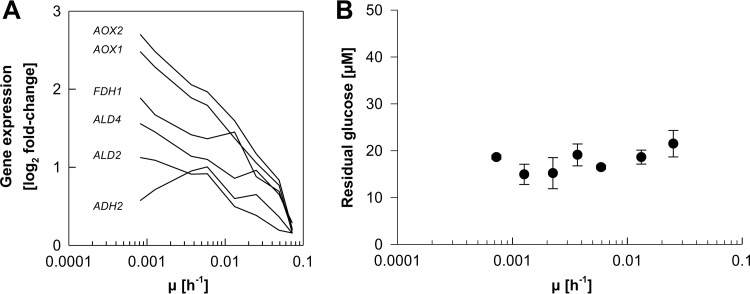
Expression profile of genes involved in alternative carbon source utilization and residual glucose concentrations in relation to the specific growth rate (μ). Shown are the expression profiles of 6 genes involved in methanol and ethanol utilization (A) and average residual glucose concentrations (B).

Corresponding to increases in the transcript levels of stress-related transcription factors toward zero growth, higher expression levels were also observed for a considerable number of other stress-responsive genes encoding, e.g., heat shock proteins (*HSP104*, *HSP31*, *HSP42*, *HSP60*, *HSP78*, *SSA3*, and *SSE1*) or proteins involved in cellular defense against oxidative stress (*CCP1-2*, *GRX3*, *MRX1-2*, *MRX2-2*, and *SOD1*). Furthermore, many genes involved in alternative carbon source utilization, whose expression is known to be repressed at elevated concentrations of glucose (20, 55), were grouped into cluster A. These genes were, for instance, genes involved in methanol (*AOX1*, *AOX2*, and *FDH1*) and ethanol (*ADH2*, *ALD2*, and *ALD4*) metabolism. Expression levels of most of these genes continued to increase with decreasing growth rates (Fig. 9A), although residual glucose levels during retentostat cultivation remained stable (Fig. 9B). Transcript levels of only one of the two high-affinity glucose transporters described for P. pastoris increased with decreasing growth rates (*HGT2*), while the expression level of the second one (*GTH1*) was strongly decreased.

**FIG 10:**
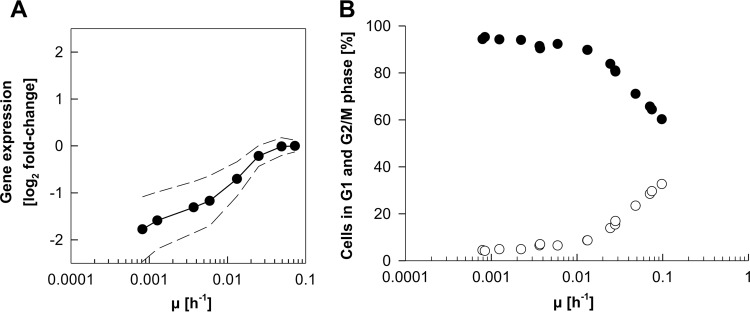
Expression profile of cell-cycle-related genes and cell cycle phase distribution of P. pastoris over a wide range of specific growth rates (μ). Shown are the average expression profile of the differentially expressed genes annotated with the GO term “cell cycle” (A) and the percentages of cells in G_1_ phase (closed circles) and G_2_/M phase (open circles) based on DNA content measurements in relation to μ (B).

Biological processes downregulated with decreasing growth rates (cluster B) (Table 2) mostly encompassed the mitotic cell cycle and related terms such as “reproduction,” “DNA metabolic process,” “chromosome segregation,” and “double-strand break repair.” In total, approximately one-fifth of all genes that were grouped into this cluster were involved in cell-cycle-related processes. The average expression levels of these cell-cycle-related genes steadily decreased toward zero growth (Fig. 10A), while the ratio between cells in G_1_, S, and G_2_/M phases changed only slightly at growth rates below 0.01 h^−1^ (Fig. 10B). Further downregulation of cell cycle-specific genes therefore cannot be attributed to shifts in cell cycle phase distribution but rather can be explained by changes within the cell cycle phase populations. For example, a decrease in the specific growth rate results in an increase in the duration of G_1_ phase. G_1_-specific cyclin expression is, however, induced just before the bud emerges (56). Consequently, with decreasing growth rates, fewer cells in G_1_ phase are in the process of passing start, resulting in an overall decrease of G_1_-specific gene expression.

Genes involved in sterol metabolism and especially in ergosterol biosynthesis (10 out of 22 genes known in P. pastoris) or transmembrane transport were also overrepresented in cluster B. The latter included genes involved in the electron transport chain, ion transport, and intra- and extracellular transport of amino acids as well as other organic compounds.

Growth-rate-related processes such as protein and nucleotide biosynthesis were not overrepresented within the differentially expressed genes, contrary to what was previously reported for producing and nonproducing P. pastoris strains at higher growth rates (7, 20). For example, out of 179 genes annotated as being involved in “ribosome biogenesis,” only 20 were significantly regulated by growth rate in the present data set, and only 12 were grouped into cluster B. Indeed, no significant changes in cellular RNA and protein contents were detected during retentostat cultivation (data not shown). Especially the unchanged RNA content is an indication that ribosome content does not change toward zero growth. Regarding amino acid biosynthesis, the expression of several genes (29 out of 119) was affected by the growth rate, with approximately half of the genes being up- and the other half being downregulated with decreasing growth rates.

The cellular contents of the storage carbohydrates glycogen and trehalose increased during retentostat cultivation (Fig. 7). The levels of both compounds reached a maximum at a specific growth rate of 0.0013 h^−1^ and decreased slightly at even lower specific growth rate. Analysis of the core set of genes involved in glycogen and trehalose metabolism (57) showed that transcript levels of these genes did not significantly change during retentostat cultivation (see Data Set S2 in the supplemental material). When transcript levels were compared to the growth rate set point of 0.1 h^−1^, upregulation toward zero growth of the genes encoding the regulatory subunit of the trehalose synthase complex (*TPS3*), the glycogen debranching enzyme (*GDB1*), and the neutral as well as the acidic trehalases (*NTH1* and *ATH1*, respectively) was significant (see Data Set S2 in the supplemental material).

Nevertheless, the absence of a clear expression trend for genes involved in storage carbohydrate metabolism during retentostat cultivation suggests that intracellular storage carbohydrate levels are not predominantly regulated at the transcriptional level. In S. cerevisiae, elements of both glycogen and trehalose metabolism are subject to strong posttranslational regulation by phosphorylation and dephosphorylation (58–61). However, to determine if a similar mechanism is responsible for the regulation of storage carbohydrate metabolism in P. pastoris, further experiments are required.

## DISCUSSION

Implementation of aerobic retentostat cultivation for P. pastoris allowed a first quantitative exploration of the physiology of this industrially relevant Crabtree-negative yeast at extremely low growth rates. Retentostat cultivation reproducibly reached biomass doubling times of over 5 weeks, corresponding to specific growth rates below 0.001 h^−1^. This domain of specific growth rates was previously explored for only one other yeast, S. cerevisiae (16, 21, 40). Similar to S. cerevisiae (16), P. pastoris exhibited increased accumulation of storage carbohydrates with decreasing specific growth rates, with combined levels of glycogen and trehalose increasing to almost 20% of the total biomass at near-zero growth rates. This increased accumulation of storage materials under severely calorie-restricted conditions may appear counterintuitive. However, from an evolutionary perspective, the allocation of scarce resources for storage can be interpreted as a typical “be prepared” scenario (62) that conditions microorganisms to adapt their physiology to multiple different scenarios, including complete depletion of energy sources. The transcriptional derepression of pathways involved in the metabolism of alternative energy substrates in the retentostat cultures of P. pastoris is also consistent with this adaptation strategy. Another similarity between P. pastoris and S. cerevisiae was the increased expression levels of genes involved in stress tolerance at low specific growth rates (21, 22), a phenomenon that has also been observed in slow-growing cultures of other microorganisms grown in retentostat cultures (13).

A first marked difference between near-zero-growth cultures of P. pastoris and S. cerevisiae concerned culture viability. Throughout the P. pastoris retentostat experiments, viability remained above 97%. Based on this high viability, an average death rate of 6 × 10^−5^ h^−1^ was calculated. This death rate is an order of magnitude lower than that observed in aerobic retentostat cultures of S. cerevisiae grown in the same range of near-zero specific growth rates (16, 21). Apparently, P. pastoris is more robust under conditions of extreme calorie restriction than S. cerevisiae. Moreover, in contrast to S. cerevisiae, which has been shown to exhibit growth-rate-independent maintenance energy requirements in anaerobic retentostat cultures (16), P. pastoris showed a sharp decline of the estimated maintenance energy requirement at near-zero specific growth rates (Fig. 6). Under these slow-growth aerobic and glucose-limited conditions, the maintenance coefficient (*m_S_*) of P. pastoris was less than half of that of corresponding cultures of S. cerevisiae (21). Its high viability and low maintenance energy requirement in aerobic retentostat cultures indicate that P. pastoris has evolved to better withstand severely calorie-restricted growth than S. cerevisiae. This conclusion is consistent with existing knowledge on the physiology and ecology of these two industrially relevant yeasts. P. pastoris, like other Crabtree-negative yeast species (63), harbors high-affinity hexose transporters (64) with glucose saturation constants that are 2 orders of magnitude lower than those of the “high-affinity” hexose transporters of S. cerevisiae (63, 65, 66). Furthermore, P. pastoris and other methylotrophic yeasts have been isolated from nutrient-poor environments such as decaying wood (67, 68). An evolutionary adaptation to energy-substrate-limited growth is also illustrated by the ability of this yeast to assimilate a wide range of carbon sources, including several nonsugars (69), and to simultaneously utilize binary mixtures of carbon sources. This has been demonstrated, among others, for glucose and methanol under limiting conditions in continuous cultures, in contrast to excess glucose, where methanol utilization is repressed (70).

To our knowledge, a growth-rate-dependent maintenance energy requirement has not been observed in a yeast or fungus so far. In S. cerevisiae, a sharp decrease of energy substrate consumption for maintenance has been reported only during energy source starvation, when metabolism becomes completely dependent on the mobilization of storage carbohydrates (15). In contrast, growth-rate-dependent maintenance energy requirements have previously been reported for several prokaryotes, for example, Escherichia coli (50), Bacillus polymyxa (49), Nitrobacter winogradskyi, and Nitrosomonas europaea (71). In these organisms, low nutrient availability activates a so-called “stringent response.” This regulatory program, triggered by the ppGpp and pppGpp alarmones, causes an upregulation of stress response genes and a downregulation of genes involved in energy-intensive processes such as protein turnover and cell division (51, 72). Further research is needed to investigate if the mechanisms responsible for reducing maintenance energy requirements in slow-growing P. pastoris cultures bear any molecular or functional resemblance to this well-characterized prokaryotic mechanism. In this respect, it is remarkable that, in contrast to observations of slow-growing and energy-starved S. cerevisiae cultures (15, 21), the retentostat cultures of P. pastoris did not show a marked transcriptional downregulation of genes involved in protein synthesis at near-zero growth rates, while expression levels of these genes correlated positively with the specific growth rate in faster-growing cultures (7). Determination of whether P. pastoris, an important cell factory for recombinant protein production, indeed has a higher translational capacity at low specific growth rates than S. cerevisiae requires further research.

Retentostat studies on P. pastoris were enabled by the construction of a *flo8* deletion mutant, which did not show dimorphism or attachment to reactor surfaces. Since putative orthologs of *FLO8* can be found in many other biotechnologically relevant yeasts, their deletion may also prove useful for bioreactor research.

### Outlook.

Yeasts are widely applied for the production of heterologous proteins and metabolites. Although physiological and molecular knowledge of P. pastoris is not as extensive as that for S. cerevisiae, it holds several advantages for aerobic bioprocess applications. P. pastoris greatly favors respiratory metabolism (23), and in contrast to the situation in S. cerevisiae, its mitochondrial respiratory chain harbors proton-translocating complex I. The present study shows that in addition, P. pastoris has very low substrate requirements for maintenance at low growth rates, a trait that is industrially relevant for the production of proteins and of metabolites whose synthesis from sugar requires a net input of ATP. Furthermore, its high tolerance to caloric restriction and extremely slow growth make P. pastoris an interesting candidate for developing zero-growth cell factory concepts. Despite the impact of maintenance energy requirements on productivity and product yields in large-scale, aerobic, fed-batch processes, very little comparative research has been done to quantify these requirements in different microorganisms. Retentostat cultivation provides a powerful tool to gain more insight into the diversity of these requirements in industrially relevant microorganisms and, ultimately, into the molecular basis for this diversity.

## Supplementary Material

Supplemental material
