# Supplementary material for: Pichia pastoris Exhibits High Viability and a Low Maintenance Energy Requirement at Near-Zero Specific Growth Rates
Source: Appl Environ Microbiol. 2016 Jul 15;82(15):4570–83. doi: 10.1128/AEM.00638-16 (PMC4984280; doi:10.1128/AEM.00638-16)
Supplement: Supplemental material [file supp_82_15_4570__index.html]

Pichia pastoris Exhibits High Viability and a Low Maintenance Energy Requirement at Near-Zero Specific Growth Rates — Supplemental material 

# Pichia pastoris Exhibits High Viability and a Low Maintenance Energy Requirement at Near-Zero Specific Growth Rates

## Supplemental material

- Supplemental file 1 -

  MATLAB codes and information on how to use these codes to perform retentostat prediction and nonlinear regression analysis

  PDF, 1.2M
- Supplemental file 2 -

  Example of retentostat data for nonlinear regression analysis (Data Set S1).

  XLSX, 209K
- Supplemental file 3 -

  Microarray data and detailed results of gene ontology term enrichment analysis for clusters A and B (Data Set S2).

  XLSX, 1.7M
