# Supplementary material for: Pichia pastoris Exhibits High Viability and a Low Maintenance Energy Requirement at Near-Zero Specific Growth Rates
Source: Appl Environ Microbiol. 2016 Jul 15;82(15):4570–83. doi: 10.1128/AEM.00638-16 (PMC4984280; doi:10.1128/AEM.00638-16)
Supplement: Supplemental material [file AEM.00638-16_zam999117303so1.pdf]

Supplemental text for:

Rebnegger C, Vos T, Graf AB, Valli M, Pronk JT, Daran-Lapujade P, Mattanovich D. *Pichia pastoris* exhibits high viability and low maintenance-energy requirement at near-zero specific growth rates

<u>Supplemental File Descriptions:</u>

Supplemental file 1 MATLAB codes and information on how to use these codes to perform retentostat prediction and non-linear regression analysis (Supplemental Methods). Apparent growth yield and maximum growth rate of the *Pichia pastoris* CBS 7435 *FLO8* and CBS 7435 *Δflo8* strain (Table S1). Viability during retentostat cultivation based on colony-forming unit count (Table S2). Regression analysis on overlapping sets of specific growth rate versus glucose uptake rate relations from 4 data points measured at different consecutive specific growth rates (Fig. S1), morphology of *P. pastoris* during retentostat cultivation (Fig. S2) and example of non-linear regression analysis of viable biomass for one retentostat (Fig. S3).

Supplemental file 2 Excel file *Example_retentostat_data.xlsx* for non-linear regression analysis.

Supplemental file 3 Excel file *Microarray_data.xlsx* containing microarray data and detailed results of GO term enrichment analysis for cluster A and B.

Supplemental Methods:

The following text provides the MATLAB codes and instructions on how to use them in order to run the retentostat prediction model and the regression model.

HOW TO – MATLAB prediction model

For the prediction analysis the MATLAB codes *Pichia_pastoris* and *Retentostat_growth* are required (see below). When converted into two separate MATLAB files (.m), these documents allow to do a prediction analysis for biomass accumulation and biomass specific rates of substrate consumption and growth in retentostat cultures. The resulting MATLAB files have to be kept in the same folder to function properly.

Pichia_pastoris.m is the MATLAB file that has to be run in MATLAB to do a prediction analysis. When executed, it calls for the function in the file *Retentostat_growth.m*. The function in the latter file contains the ordinary differential equations (ODEs) that are needed to describe biomass accumulation and corresponding rates of growth and substrate consumption in the retentostat (see Materials and Methods). These ODEs are solved multiple times for varying values of m_S (maintenance energy requirements m_S), and V_A (mixing vessel).

Data are plotted in MATLAB and presented as three dimensional plots depicting on the different axis: biomass concentration, the maintenance coefficient, and the volume of the mixing vessel. A final plot depicts the growth kinetics in retentostat for a minimum and maximum value of m_S and a chosen working volume in the mixing vessel described in the command window after the prediction is finished, shown below in red:

"The maximal volume of mixing vessel A for which a robust set-up can be guaranteed based on this model, up to an m_S of 0.012 is a volume of 1.3 L."

To personalize the files for prediction of new retentostat experiments with other organisms or different conditions, one can change the values in the code *Pichia_pastoris*:

```
V_B = 1.4; % L (Bioreactor working volume)
Cs_chem = 20 ; % g/L (glucose concentration in medium reservoir during chemostat)
Cs_ret = 10 ; % g/L     (glucose concentration in medium reservoir during retentostat)
Ysx_max = 0.65; % g/g          (maximum biomass yield on glucose)
```

```
Ysp_max = 0.168; % g/g        (maximum product yield on glucose) Must be a non-zero value!
ms_est = 0.0135; % gs/gx/h    (initial estimate of the maintenance coefficient)
D_chem = 0.025; % h-1         (dilution rate)
F_in = V_B*D_chem; % L/h      (medium flow rate)
days = 30; % d                (retentostat operation time)
```

Optional product formation terms can be included as well: for linear qp (mu)-relations (qp = a*mu+b) qp_func = parameter a and qp_res = parameter b, with qp [$mol \cdot g_X^{-1} \cdot h^{-1}$] and Ysp_max in [$mol_P \cdot mol_S^{-1}$]. For non-producing strains qp terms equal 0:

```
% for linear qp(mu)-relations qp = a*mu+b
qp_func = 0; % parameter a
qp_res = 0; % parameter b
```

In the code *Retentostat_growth*, values for volume of the mixing vessel (V_A) are described according to:

```
for p = 1:maxp
   %% Process Parameters
   % Vessel A - Mixing Vessel
   %     Fin_A = 0.035; % L/h
   V_A = 0 + p*0.1; %L
```

and range between 0 and 2 L.

In the code *Retentostat_growth*, values for the maintenance requirements (ms) are described according to:

```
ms = (ms_est-maxi/2*0.0001) + i*0.0001 % maintenance coefficient % gs/gx/h
```

and thereby allows calculations for a range of mS values around ms_est with the code *Pichia_pastoris*.

Pichia_pastoris

```
function Pichia_pastoris
clear all
close all
clc
format compact


Strain = ['Pichia'];
Strain


V_B = 1.4; % L
Cs_chem = 20 ; % g/L
Cs_ret = 10 ; % g/L
Ysx_max = 0.65; % g/g
Ysp_max = 0.168; % g/g
ms_est = 0.0135; % gs/gx/h
D_chem = 0.025; % h-1
F_in = V_B*D_chem; % L/h
days = 30; % days

% Production (if there is production, remove "%" and qp_func is not 0)
%D = [0;0.015; 0.025; 0.050; 0.075; 0.100; 0.125;0.150]; % 1/h
%qp_LAB = [0;0.008; 0.035; 0.055; 0.063; 0.078; 0.110; 0.174]/1000; % g/g/h

%qp_func = (qp_LAB(3)/D(3));
 qp_func = 0;
 qp_res = 0;

retentostat_growth(V_B,Cs_chem, Cs_ret, Ysx_max, Ysp_max, ms_est, F_in, days, qp_func,
qp_res);



end
```

Retentostat_growth

```
function retentostat_growth(V_B,Cs_chem, Cs_ret, Ysx_max, Ysp_max, ms_est, F_in, days,
qp_func, qp_res)

% By putting two percent-signs (%%), one can mark parts of the code.
%% Looping over the volume of the mixing vessel (Vessel A)

maxp = 20; % iterations over the volume of vessel A
maxi = 20; % iterations over the maintenance coefficient.
Fin_A = F_in;

Cx_end = zeros(maxp,maxi);
Cx_max = zeros(maxp,maxi);
V_Ass = [];
mu = [];

% Vectors for the final analysis, storing ALL datapoints over time
Cx_t = [];
mu_t = [];
Cs_t = [];
Cp_t = [];

for p = 1:maxp
    %% Process Parameters
    % Vessel A - Mixing Vessel
    %     Fin_A = 0.035; % L/h
    V_A = 0 + p*0.1; %L
    Fout_A = Fin_A; % L/h

    % Vessel B - Fermenter
    Fin_B = Fout_A;
    Fout_B = Fin_B;

    %% Storage matrices
    % Because of the loops used in this function, certain values are
    % required to be stored in matrices. When an ode-solver is in a loop,
    % the output will usually be stored in the output matrix (c in this
    % function), but will be overwritten once the loop is run again with a
    % new value. This would mean a loss of data if the data wouldn't be
    % stored in a 'storage matrix'. this is necessary when the data needs
    % to be accessed later.

    Cx = []; % storage matrix for biomass concentrations in vessel B
    Cs = []; % storage matrix for substrate concentrations in vessel A
    Cp = []; % storage matrix for product concentrations in vessel B
    mss = []; % Storage matrix for the ms-values (for the plotting)
    tss= []; % storage vector for the time-points (for plotting)
```

```
%% Time span of the ode-solver in h

tini= 0 ; % h
tend= 24*days; % h
dt  = 0.5; % the time steps that are used for the ode solver, [h]

%% Initial concentrations for the ode-solver
% In this model, these are the values that are obtained from the
% chemostat phase. The time of 0 hours is when the chemostat is
% switched to a retentostat.

C_S0 = Cs_chem; %  g/L Substrate in the inflow of vessel A

%% Looping over ms

% Herbert Pirt Equation Parameters for product formation
A = qp_func;
B = (Ysp_max+Ysx_max*A)/(Ysx_max*Ysp_max);
G = qp_res/Ysp_max;

for i = 1:maxi

    ms = (ms_est-maxi/2*0.0001) + i*0.0001 % maintenance coefficient % gs/gx/h

    C_X0 = ((Fin_B/V_B)*C_S0)/(((Fin_B/V_B)*B)+ms + G);
    C_P0 = (A*(Fin_B/V_B)+qp_res)*C_X0*V_B/Fout_B;
    c0 = [C_X0 C_S0 C_P0];% C_SB0];

    %% ode solver
    opt = odeset('RelTol',1e-8, 'AbsTol',1e-8);
    [t,c] = ode45(@(t,c)dcdt(t,c,ms,Ysx_max,Ysp_max,Cs_ret,F_in,V_A,V_B, qp_res, B, A, G),
tini:dt:tend, c0, opt);

    % there are several ode solvers available. ode45 is most common to
    % use for 'simple' problems

    % In order to keep the data available outside of the loop, the concentrations that are
    % yielded from the ode solver, they are stored in the
    % storage-matrices as defined above.

    Cx = [Cx c(:,1)]; Cx_t = [Cx_t c(:,1)];
    Cs = [Cs c(:,2)]; Cs_t = [Cs_t c(:,2)];
    Cp = [Cp c(:,3)]; Cp_t = [Cp_t c(:,3)];
    Cs_B = 0;

    %% Plotting & Data Handling
```

```
        % Calculate mu, qp, qs, Ysp qs(mu) and qs(ms) are calculated from the Herbert-pirt
equation.
        for k = 1:1:length(t)
           qs(k,i) = ((Fin_B/V_B)*(Cs(k,i)-Cs_B))/Cx(k,i);
           qs_t(k,i+(p-1)*maxi) = qs(k,i);
           mu(k,i) = (qs(k,i)-G-ms)/B;  % based on substrate balance
           mu_t(k,i+(p-1)*maxi) = mu(k,i);
           qp(k,i) = A*mu(k,i)+qp_res;
           qp_t(k,i+(p-1)*maxi) = qp(k,i);
           Ysp(k,i) = qp(k,i)/qs(k,i);
           Ysp_t(k,i+(p-1)*maxi) = Ysp(k,i);
           qs_mu(k,i) = (mu(k,i)/Ysx_max)/qs(k,i);
           qs_mu_t(k,i+(p-1)*maxi) = qs_mu(k,i);
           qs_ms(k,i) = ms/qs(k,i);
           qs_ms_t(k,i+(p-1)*maxi) = qs_ms(k,i);
        end

        % as soon as the new loop starts, the data in matrices t and c and
        % the value for ms will be overwritten. Therefore, that data needs
        % to be stored in a nother matrix, that will contain all data of
        % all the loops.

        Cx_end(p,i) = c(end,1);
        Cx_max(p,i) = max(c(:,1));
        mss = [mss;ms];
        tss = [tss t];

    end

    V_Ass = [V_Ass V_A];

    %% Final data plotting


    figure(p*10)
    surf(mss,tss/24,Cx,'EdgeColor','none');
    xlabel(' m_s (g_{glucose}.g_x^{-1}.h^{-1})', 'FontSize',14 );
    ylabel('Time (d)', 'FontSize',14 );
    zlabel('C_x (g.L^{-1})', 'FontSize',14 );
    suptitle(['Volume_A = ' num2str(V_Ass(end)) ' L'])
    %when writing strings (of letters), an underscore (_) gives the next
    %letter as subscript OR whatever is between braces: {}

end

%close all %closes small plots before final data plotting
```

```
%% Function Final Output

% The function should give the required answers on maximal volume of vessel A for
maximal ms
% values as written output in the command window. to give written output,
% the commands fprintf are used. The constraint on Volume of vessel A is
% defined by the maximal volume for which the biomass specific growth rate
% remains non-negative (= starvation).
Cx_eval = Cx_max./Cx_end; % evaluate whether Cx max is at the end of the
% retentostat
mu_thres = ones(length(t),1)*1e-3; % This vector is created to have a
% threshold line for the growth rate (has to be below 0.001 h-1

for p = 1:maxp;
    if Cx_eval(p,maxi) > 1
        % It is easiest to have the output in an easily accessible format,
        % here a sentence.
        fprintf(['The maximal volume of mixing vessel A for ' ...
            'which a robust set-up can be guaranteed \n based on this model, ' ...
            'up to an ms of ' num2str(mss(maxi)) ' is a volume of '...
            num2str(V_Ass(p-1)) ' L. \n' ])
        % It has to be p-1, because if p is used for the volume, the Cx_eval
        % is already > 1.

        % but a visual check of the conclusion is required as well.
        for a = 0:1 % plot for the first 'wrong'  V_A and the last ' good' V_A.

            figure((p-a)*101);% suptitle(['Volume_A = ' num2str(V_Ass(p-a)) ' L'])
            Fontsize = 15;
            % Biomass concentration
            subplot(3,3,1)
            plot(t/24,Cx_t(:,1+((p-a)-1)*maxi),'r',t/24,Cx_t(:,(maxi+((p-a)-1)*maxi)),'k-',
'LineWidth', 1.5);
            hold on;
            xlabel(' Time (d)', 'FontSize',15 );
            ylabel('C_x (g.L^{-1})', 'FontSize',15 );
            title('a','FontSize',Fontsize);
            % Specific growth rate
            subplot(3,3,4)
            plot(t/24,mu_t(:,1+((p-a)-1)*maxi),'r',t/24,mu_t(:,maxi+((p-a)-1)*maxi),'k-',
'LineWidth', 1.5);
            hold on;
            xlabel(' Time (d)', 'FontSize',15  );
            ylabel('\mu (h^{-1})', 'FontSize',15 );
            title('d','FontSize',Fontsize);
            % Substrate inflow concentration
            subplot(3,3,2)
```

```
plot(t/24,Cs_t(:,1+((p-a)-1)*maxi),'r',t/24,Cs_t(:,maxi+((p-a)-1)*maxi),...
    'k-', 'LineWidth', 1.5);
hold on;
xlabel(' Time (d)', 'FontSize',15  );
ylabel('C_{s,}_{in} (g.L^{-1})', 'FontSize',15 );
title('b','FontSize',Fontsize);
% Specific growth rate (logarithmic scale)
subplot(3,3,7)
semilogy(t/24,mu_t(:,1+((p-a)-1)*maxi),'r',t/24,mu_t(:,maxi+((p-a)-1)*maxi),...
    'k-',t/24,mu_thres,'g', 'LineWidth', 1.5);
hold on;
xlabel(' Time (d)', 'FontSize',15  );
ylabel('\mu (h^{-1})', 'FontSize',15 );
title('g','FontSize',Fontsize);
% specific product formation rate
subplot(3,3,6)
plot(t/24,qp_t(:,1+((p-a)-1)*maxi),'r',t/24,qp_t(:,maxi+((p-a)-1)*maxi),...
    'k-', 'LineWidth', 1.5);
hold on;
xlabel(' Time (d)', 'FontSize',15  );
ylabel('q_p (g_{p}.g_{x}^{-1}.h^{-1})', 'FontSize',15 );
title('f','FontSize',Fontsize);
% product concentration
subplot(3,3,3)
plot(t/24,Cp_t(:,1+((p-a)-1)*maxi),'r',t/24,Cp_t(:,maxi+((p-a)-1)*maxi),...
    'k-', 'LineWidth', 1.5);
hold on;
xlabel(' Time (d)', 'FontSize',15  );
ylabel('C_p (g.L^{-1})', 'FontSize',15 );
title('c','FontSize',Fontsize);
% specific substrate consumption rate
subplot(3,3,5)
plot(t/24,qs_t(:,1+((p-a)-1)*maxi),'r',t/24,qs_t(:,maxi+((p-a)-1)*maxi),...
    'k-', 'LineWidth', 1.5);
hold on;
xlabel(' Time (d)', 'FontSize',15  );
ylabel('q_s (g_{s}.g_x^{-1}.h^{-1})', 'FontSize',15 );
title('e','FontSize',Fontsize);
axis([0,1,0 , 0.07], 'auto x')

subplot(3,3,8)
plot(t/24,qs_mu_t(:,1+((p-a)-1)*maxi)*100,'r',t/24,qs_ms_t(:,1+((p-a)-1)*maxi)*100,
'k','LineWidth', 1.5);
hold on
plot(t/24,qs_mu_t(:,maxi+((p-a)-1)*maxi)*100,'g',t/24,qs_ms_t(:,maxi+((p-a)-
1)*maxi)*100, 'b','LineWidth', 1.5);
xlabel(' Time (d)', 'FontSize',Fontsize );
```

```
        ylabel('Gluc. distr. (%)', 'FontSize',Fontsize );
        title('h','FontSize',Fontsize );


        % Product on substrate yield
        subplot(3,3,9)
        plot(t/24,Ysp_t(:,1+((p-a)-1)*maxi),'r',t/24,Ysp_t(:,maxi+((p-a)-1)*maxi),...
            'k-', 'LineWidth', 1.5);
        hold on;
        xlabel(' Time (d)', 'FontSize',15  );
        ylabel('Y_{sp} (g_p.g_s^{-1})', 'FontSize',15 );
        title('i','FontSize',Fontsize);


      end

      break

    end
  end


  end

function dc = dcdt(t,c,ms,Ysx_max,Ysp_max,Cs_ret,F_in,V_A,V_B, qp_res, B, A, G)
% global Fin_A V_A Fout_A ms Ysx_max Ysp_max qp V_B


%% Variables
Cx_B = c(1);
Cs_A = c(2);
Cp_B = c(3);
Cs_B = 0;

%% Model Parameters

% Vessel A - Mixing Vessel

Cs_R = Cs_ret; % the concentration in the second medium vessel; eventually the
concentration in vessel A.

% Vessel B - Fermentor
Fin_A = F_in;
Fout_A = Fin_A;
Fin_B = Fout_A;
Fout_B = Fin_B;
```

```
qs = ((Fin_B/V_B)*(Cs_A-Cs_B))/Cx_B;
mu = (qs - G - ms)/B;
qp = A * mu + qp_res;

%% Equations
dc(1) = mu*Cx_B; % Balance of Cx_B
dc(2) = Fin_A/V_A*Cs_R - Fout_A/V_A*Cs_A; %  % Balance of Cs_A
dc(3) = qp*Cx_B-Fout_B*Cp_B; % Balance of Cp_B: Product formation

dc = dc';
end
```

HOW TO – MATLAB regression model

For the non-linear regression analysis the MATLAB codes *Example_rententostat_data, Retentostat_regression_Pp_kd* and_*plotting* (see below) as well as the Excel supplemental file 2 (*Example_retentostat_data.xlsx)* are required. The MATLAB codes need to be converted into separate MATLAB files. Together, these documents allow to perform a regression analysis on experimental data from retentostat cultures, and have to be kept in the same folder to function properly.

Excel sheet *MATLABread* in the file *Example_retentostat_data.xlsx*, contains data collected from an example retentostat experiment. In this sheet are incorporated the (viable) biomass concentration and the glucose concentration in the feed. The different volumes and flow rates taken into account by the model are indicated in the data sheet as well.

Example_rententostat_data is the MATLAB code that has to be run in MATLAB to do a regression analysis. When executed, it automatically requests data from Sheet *MATLABread* in the file *Example_retentostat_data.xlsx*, and calls for the function in the code *Retentostat_regression_Pp_kd*. The function in the latter contain the ordinary differential equations (ODEs) that are needed to describe (viable and total) biomass accumulation in the retentostat (see Materials and Methods), which are solved multiple times for varying values of m_S (maintenance energy requirements m_S), and k_d (death rate k_d) until the sum of square errors (SSE) is minimized.

Data is plotted using the code *retentostat_plotting*.

Sheet *Modelling Summary* in the Excel file *Example_retentostat_data.xlsx* is where MATLAB deposits the data after a complete regression analysis. In here, ms opt is the value for m_S (in $mg \cdot g^{-1} \cdot h^{-1}$) corresponding to the regression with the lowest SSE. In addition, values for SSE, r-squared (Rsq), optimised death rate (Kd [“value”$\cdot 10^{-3}$ h^{-1}]) and the final specific growth rate at the end of retentostat (in h^{-1}). Furthermore, the glucose concentration in the feed (Cs), the biomass concentration in the retentostat (Cx) the specific growth rate (mu) and the specific substrate uptake rate (qs) are published according to the regression analysis, with a step size of 30 minutes.

To personalize the files for new retentostat experiments with other organisms or different conditions, one can change the following values, arranged according to the files they appear in:

Code *Example_rententostat_data.xlsx*

- Change all data in sheet *MATLABread* according to your personal experimental data
- Sheet *Modelling Summary* is automatically emptied and filled with each new regression analysis.

Code *Example_rententostat_data*

- Yeast parameters (qp and Ysp_max refers to production characteristics of a strain*)
- The data location that is read by MATLAB from an Excel file (`filename = 'Example_retentostat_data.xlsx'`).

File *Retentostat_regression_Pp_kd*

- Initial guesses for ms and kd (in $g_{glucose} \cdot g_{biomass}^{-1} \cdot h^{-1}$ and in h^{-1}, respectively), starting point for solving ODEs and minimizing SSE.
- Time span of retentostat (in days)
- Concentration in the medium reservoir during retentostat

*Optional product terms: for linear qp (mu)-relations (qp = a*mu+b), qp_func = parameter a and qp_res = parameter b, with qp [$g \cdot g_X^{-1} \cdot h^{-1}$] and Ysp_max in [$g_P \cdot g_S^{-1}$]. For non-producing strains qp terms equal 0.

Retentostat_regression_Pp_kd

```
function [ms_opt,kd_opt, SSE, Cx, Cs, mu, qs, t, Rsq, mu_end, t_dub] =
Retentostat_regression_Pp_kd(TIME_CSin, CS_in_LAB, Fin_A,V_A, V_B, Ysx_max, Ysp_max,
qp_func, qp_res, CX_VIAB, TIME_LAB_V, CX_LAB_std_V, filename, DW, DW_std);

%% Global Parameters
global TIME_LAB_V CX_VIAB TIME_CSin CS_in_LAB V_A V_B Fin_A A B CX_LAB_std_V
filename G qp_res Ysx_max DW DW_std


TIME_LAB_V
%% Process Parameters
% Vessel A - Mixing Vessel
Fout_A = Fin_A; % L/h
% Vessel B - Fermenter
Fin_B = Fout_A;

A = qp_func;
B = (Ysp_max+Ysx_max*A)/(Ysx_max*Ysp_max);
G = qp_res/Ysp_max;

%% Optimisation function
%p0 = [0.008]; % initial guess
x0 = [0.0008;
    0.00001]; ... kd


options = optimset('TolFun',1e-7,'TolX',1e-7,'Display','iter','MaxIter',1000,
'PlotFcns',@optimplotx);    % note: to display how par
[x,fval,exitflag,output] = fminsearch(@funobj,x0,options);

format short
ms_opt = x(1)*1000; % mg/gx/h
kd_opt = x(2)*1000; % h-1
SSE = fval;

%% Time span of the ode-solver in h
days = 25; % days the retentostat will run

tini= 0 ; % h
tend= 24*days; % h
dt  = 0.5; % the time steps that are used for the ode solver.

% Merge all measured times into one vector and select unique times:
tspan = [tini:dt:tend];
TIME_LAB_V_h = TIME_LAB_V*24;
tspan = [tspan'; TIME_LAB_V_h];
```

```
tspan = sort(unique(tspan));        % this is the entire tspan, for all datapoints


C_S0 = CS_in_LAB(1);  %  g/L Substrate in the inflow of vessel A
C_X0 = CX_VIAB(1);
C_P0 = 0;
C_Xd0 = DW(1) - CX_VIAB(1);

c0 = [C_X0 C_S0 C_P0 C_Xd0];
ms = x(1);
kd = x(2);

%% ode solver
opt = odeset('RelTol',1e-8, 'AbsTol',1e-8);
[t,c] = ode45(@(t,c)dcdt(t,c,ms,kd), tspan, c0, opt);

% storage-matrices
Cx = c(:,1);
Cs = c(:,2);
Cp = c(:,3);
Cxd = c(:,4);
Cdw = Cx + Cxd;

SSE_X = sum(((c(find(ismember(tspan, TIME_LAB_V_h)),1) - (CX_VIAB))./CX_LAB_std_V).^2)
SSE_tot = sum(((c(find(ismember(tspan, TIME_LAB_V_h)),1) -
(mean(CX_VIAB)))./CX_LAB_std_V).^2);

SSE_Xd = sum(((c(find(ismember(tspan, TIME_LAB_V_h)),4) - (DW-CX_VIAB))./DW_std).^2);
SSE_totd = sum(((c(find(ismember(tspan, TIME_LAB_V_h)),4) - (mean(DW-
CX_VIAB)))./DW_std).^2);


% This value should be optimized
SSE_total = SSE_X + SSE_Xd;


Rsq = 1-(SSE_X/SSE_tot)
Rsqd = 1-(SSE_Xd/SSE_totd)

Cs_B = 0;

% Calculate mu
% mu is calculated from the Herbert-pirt equation.
% Based on this mu, the qs value is calculated

for k = 1:1:length(t)
   qs(k) = (Fin_B/V_B*(Cs(k)-Cs_B))/Cx(k);
```

```
    mu(k) = (qs(k)-G-ms)/B;
    qp(k) = A*mu(k)+qp_res;

    Ysp(k) = qp(k)/qs(k);
    qs_mu(k) = (mu(k)/Ysx_max)/qs(k);
    qs_ms(k) = ms/qs(k);
end

mu = mu';
qs = qs';

Figno = 102;
SAVE_on = 1;
retentostat_plotting(t,Cx,Cs,Cp,mu,qs,qp,Ysp, qs_mu, qs_ms, Figno, V_A, TIME_LAB_V,
CX_VIAB, TIME_CSin, CS_in_LAB, SAVE_on, filename, ms_opt, DW, Cdw)

mu_end = mu(end);
t_dub = log(2)/mu_end;

end

function [SSE_total, Cx] = funobj(p)
%% Global definition of parameters
global Fin_A V_A Fout_A Ysx_max  qp V_B TIME_LAB_V  CX_VIAB TIME_CSin CS_in_LAB  A B
CX_LAB_std_V filename G qp_res DW DW_std Cs_R

%% Process Parameters
% Vessel A - Mixing Vessel
Fout_A = Fin_A; % L/h

% Vessel B - Fermenter
Fin_B = Fout_A;
% Ysx_max = 0.5; % g/g

%% Time span of the ode-solver in h

days = 25; % days the retentostat will run

tini= 0 ; % h
tend= 24*days; % h
dt  = 0.5; % the time steps that are used for the ode solver.
TIME_LAB_V;

% Merge all measured times into one vector and select unique times:
tspan = [tini:dt:tend];
TIME_LAB_V_h = TIME_LAB_V*24;
tspan = [tspan'; TIME_LAB_V_h];
```

```
tspan = sort(unique(tspan));        % this is the entire tspan, for all datapoints


%% Initial concentrations for the ode-solver
% In this model, these are the values that are obtained from the
% chemostat phase, where we know there is a steady state. The time of 0 hours is when the
chemostat is
% switched to a retentostat.

C_S0 = CS_in_LAB(1); %  g/L Substrate in the inflow of vessel A
C_P0 = 0; %  g/L Product in the outflow of vessel B
C_Xd0 = 0;

ms = p(1); % maintenance coefficient % gs/gx/h
kd = p(2);

C_X0 = CX_VIAB(1);
C_Xd0 = DW(1)-CX_VIAB(1);

c0 = [C_X0 C_S0 C_P0 C_Xd0];
Cs_R = CS_in_LAB(end);
%% ode solver
opt = odeset('RelTol',1e-8, 'AbsTol',1e-8);
[t,c] = ode45(@(t,c)dcdt(t,c,ms,kd, Cs_R), tspan, c0, opt);

% storage-matrices
Cx = c(:,1);
Cs = c(:,2);
Cp = c(:,3);
Cxd = c(:,4);
Cdw = Cx + Cxd;

%% Plotting & Data Handling

% Calculate mu
% mu is calculated from the Herbert-pirt equation.
% Based on this mu, the qs, qp and Ysp values are calculated
Cs_B = 0;
for k = 1:length(t)
   qs(k) = (Fin_B/V_B*(Cs(k)-Cs_B))/Cx(k);
   mu(k) = (qs(k)-G-ms)/B;
   qp(k) = A*mu(k)+qp_res;
   Ysp(k) = qp(k)/qs(k);
   qs_mu(k) = (mu(k)/Ysx_max)/qs(k);
   qs_ms(k) = ms/qs(k);
end
```

```
%% Final data plotting


%% Function Final Output
% Select ODE values for the right tspan values and calculate sum of squares
% format long
c(find(ismember(tspan, TIME_LAB_V_h)),1)
(CX_VIAB)./CX_LAB_std_V


SSE_X = sum(((c(find(ismember(tspan, TIME_LAB_V_h)),1) - (CX_VIAB))./CX_LAB_std_V).^2)
SSE_tot = sum(((c(find(ismember(tspan, TIME_LAB_V_h)),1) -
(mean(CX_VIAB)))./CX_LAB_std_V).^2)

SSE_Xd = sum(((c(find(ismember(tspan, TIME_LAB_V_h)),4) - (DW-CX_VIAB))./DW_std).^2)
SSE_totd = sum(((c(find(ismember(tspan, TIME_LAB_V_h)),4) - (mean(DW-
CX_VIAB)))./DW_std).^2)

R2v = 1-(SSE_X/SSE_tot);
R2d = 1-(SSE_Xd/SSE_totd);

% This value should be optimized
SSE_total = SSE_X + SSE_Xd;

Figno = 101;
SAVE_on = 0;
%retentostat_plotting(t,Cx,Cs,Cp,mu,qs,qp,Ysp, qs_mu, qs_ms, Figno, V_A, TIME_LAB_V,
CX_VIAB, TIME_CSin, CS_in_LAB, SAVE_on, filename, ms*1000, DW, Cdw)

end

function dc = dcdt(t,c,ms,kd, Cs_R)
global Fin_A V_A V_B A B G qp_res

%% Variables
Cx_B = c(1);
Cs_A = c(2);
Cp_B = c(3);
Cxd_B = c(4);
Cs_B = 0;


%% Model Parameters

% Vessel A - Mixing Vessel
Cs_R = 5; % the concentration in the second medium vessel; eventually the concentration in
vessel A.
```

```
% Vessel B - Fermentor
Fout_A = Fin_A;
Fin_B = Fout_A;
Fout_B = Fin_B;

qs = ((Fin_B/V_B)*(Cs_A-Cs_B))/Cx_B;
mu = (qs - G - ms)/B;
qp = A * mu + qp_res;

%% Equations
dc(1) = mu*Cx_B - kd*Cx_B; % Balance of Cx_B
dc(2) = Fin_A/V_A*Cs_R - Fout_A/V_A*Cs_A; %  % Balance of Cs_A
dc(3) = qp*Cx_B-Fout_B*Cp_B; % Balance of Cp_B: Product formation
dc(4) = kd * Cx_B; % balance of dead biomass

dc = dc';
end
```

Retentostat_plotting

```
function retentostat_plotting(t,Cx,Cs,Cp,mu,qs,qp,Ysp, qs_mu, qs_ms, Figno, V_A,
TIME_LAB_V, CX_VIAB, TIME_CSin, CS_in_LAB, SAVE_on, filename, ms, DW, Cdw)

h = figure(Figno);
if SAVE_on == 0
suptitle(['Volume_A = ' num2str(V_A) ' L, ms = ' num2str(ms) ' mg/gx/h ' ])
end
Fontsize = 13;
Linewidth = 1;

subplot(3,3,1)
plot(t/24,Cx(:,1),'k-.', 'LineWidth', Linewidth);
hold on;
plot(t/24,Cdw(:,1), 'r-.', 'LineWidth', Linewidth);
hold on
xlabel(' Time (d)', 'FontSize',Fontsize );
ylabel('C_x (g.L^{-1})', 'FontSize',Fontsize );
hold on;
plot(TIME_LAB_V, CX_VIAB,'m.','LineWidth',Linewidth);
hold on
plot(TIME_LAB_V, DW,'g.', 'LineWidth', Linewidth);
title('a','FontSize',Fontsize );
axis([0,25,0 , 1], 'auto y')

subplot(3,3,2)
plot(t/24,Cs,'k-.', 'LineWidth', Linewidth);
hold on;
xlabel(' Time (d)', 'FontSize',Fontsize  );
ylabel('C_{s,}_{in} (g.L^{-1})', 'FontSize',Fontsize );
plot(TIME_CSin, CS_in_LAB,'.')
title('b','FontSize',Fontsize );
axis([0,25,0 , 1], 'auto y')

subplot(3,3,3)
plot(t/24,Cp, 'k','LineWidth', Linewidth);
xlabel(' Time (d)', 'FontSize',Fontsize  );
ylabel('C_p (g_{p}.L^{-1})', 'FontSize',Fontsize );
title('c','FontSize',Fontsize );
axis([0,25,0 , 1], 'auto y')


subplot(3,3,4)
plot(t/24,mu,'k-.', 'LineWidth', Linewidth);
hold on;
xlabel(' Time (d)', 'FontSize',Fontsize  );
ylabel('\mu (h^{-1})', 'FontSize',Fontsize );
```

```
title('d','FontSize',Fontsize );
axis([0,25,0 , 1], 'auto y')

subplot(3,3,5)
plot(t/24,qs, 'k','LineWidth', Linewidth);
hold on
plot(t/24,ones(length(t))*ms/1000,'g', 'LineWidth',Linewidth);
xlabel(' Time (d)', 'FontSize',Fontsize  );
ylabel('q_s (g_{s}.g_x^{-1}.h^{-1})', 'FontSize',Fontsize );
title('e','FontSize',Fontsize );
axis([0,25,0 , 1], 'auto y')

subplot(3,3,6)
plot(t/24,qp, 'k','LineWidth', Linewidth);
xlabel(' Time (d)', 'FontSize',Fontsize  );
ylabel('q_p (g_{p}.g_x^{-1}.h^{-1})', 'FontSize',Fontsize );
  title('f','FontSize',Fontsize );
  axis([0,25,0 , 1], 'auto y')

subplot(3,3,7)
semilogy(t/24,mu,'m', 'LineWidth', Linewidth);
hold on;
xlabel(' Time (d)', 'FontSize',Fontsize  );
ylabel('\mu (h^{-1})', 'FontSize',Fontsize );
title('g','FontSize',Fontsize );
axis([0,25,0 , 1], 'auto y')

subplot(3,3,8)
plot(t/24,qs_mu*100,t/24,qs_ms*100, 'k','LineWidth', Linewidth);
xlabel(' Time (d)', 'FontSize',Fontsize  );
ylabel('Gluc. distr. (%)', 'FontSize',Fontsize );
title('h','FontSize',Fontsize );
axis([0,25,0 , 1], 'auto y')

subplot(3,3,9)
plot(t/24,Ysp, 'k','LineWidth', Linewidth);
xlabel(' Time (d)', 'FontSize',Fontsize  );
ylabel('Y_{sp} (g_{p}.g_{s}^{-1})', 'FontSize',Fontsize );
title('i','FontSize',Fontsize );
axis([0,25,0 , 1], 'auto y')

if SAVE_on == 1
   saveas(h,[num2str(date) num2str(filename(1:end-4)) '_final_plot'],'m')
end

end
```

Supplemenal Figures and Tables:

TABLE S1 Apparent biomass yield ($Y_{X/S}$) and maximum specific growth rate (μ_{max}) of wild-type *P. pastoris* CBS 7435 (*FLO8*) and an isogenic *Δflo8* strain.

Strain	YX/S* (± SD)	μmax (± SD)
CBS 7435 *Δflo8*	0.511 ± 0.0045	0.22 ± 0.0013
CBS 7435 *FLO8*	0.534 ± 0.0030	0.25 ± 0.0026

*Values for $Y_{X/S}$ were derived from aerobic glucose limited chemostat cultivations operated at a dilution rate of 0.05 h^{-1}. The $Y_{X/S}$ for the CBS 7435 *FLO8* is based on one biological replicate.

TABLE S2 Colony-forming units (CFU)* in percent of total cell concentration during retentostat cultivation.

Days after switch to retentostat mode	CFU for reactor 1 (%)	CFU for reactor 2 (%)
18.8	139 ± 1.3	106 ± 8.0
22.7	91 ± 3.4	92 ± 4.6
23.7	103 ± 6.7	85 ± 5.1

*CFU was assessed by diluting cells in sterile 0.1 % peptone to approximately 5×10^7 cells per mL and sonicated at 8 ampere for 3 seconds (Soniprep 150, Fisher Scientific, USA). The exact cell concentration was determined using a hemocytometer and tenfold dilution series were made (5 to 6 dilutions) in 0.1 % peptone. Appropriate aliquots of the final dilutions were plated on YPD plates in at least 2 technical duplicates and incubated at 30 °C for two days before colonies were counted.

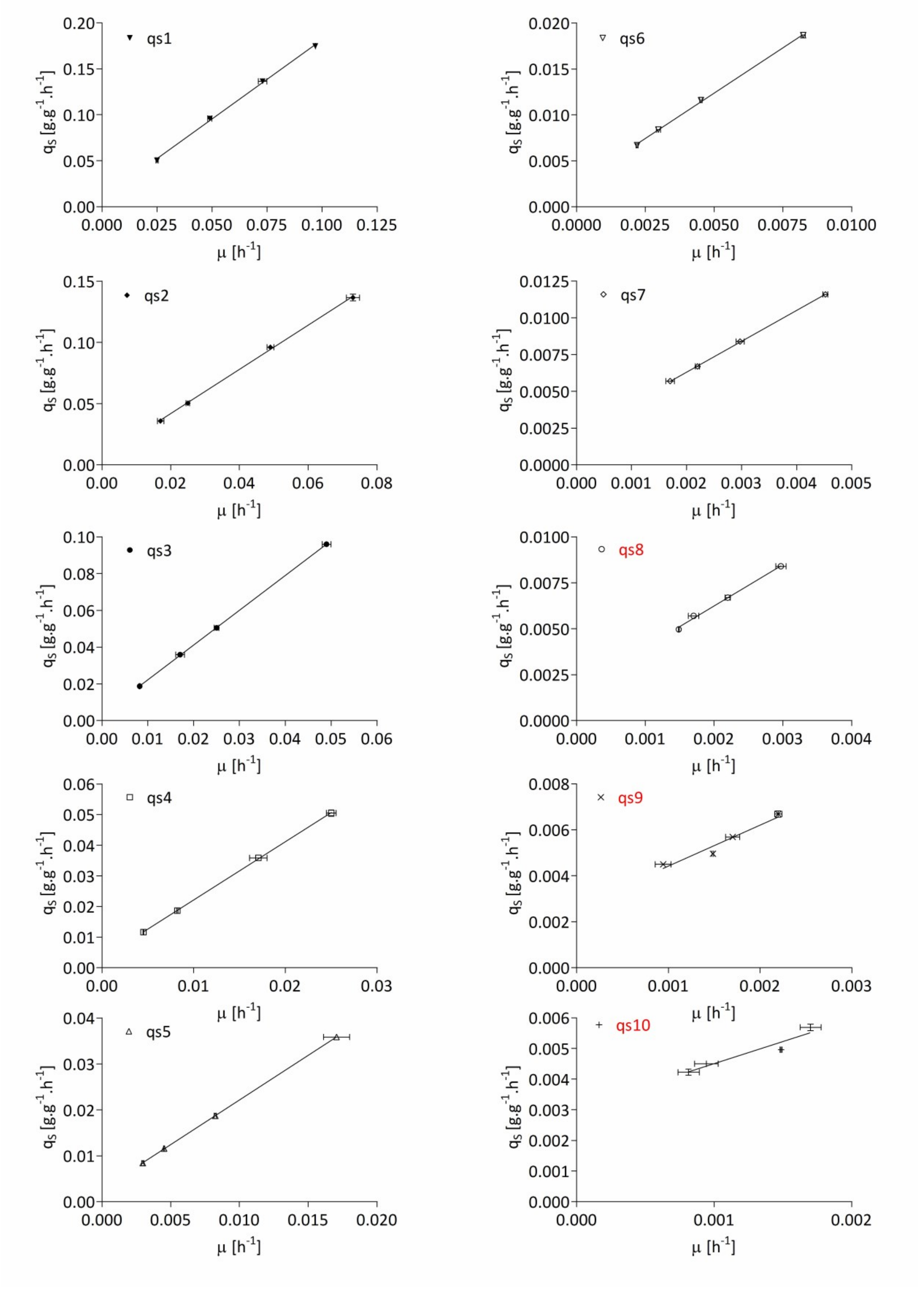

FIG S1 Regression analysis on overlapping sets of specific growth rate versus glucose uptake rate relations (μ - q_S) from 4 data points measured at different consecutive specific growth rates (see Materials and Methods). Values for the maintenance coefficient (m_S) and the maximum yield on biomass ($Y_{X/S}^{max}$) were calculated from plots q_S1 - q_S7 which resulted in a

R2 of at least 0.99. Linear regression on data represented in plots q_S8 - q_S10 resulted in R2 values below 0.99 and were not considered further to assess the growth rate dependency of m_S and $Y_{X/S}^{max}$ as depicted in **Fig 6** in the manuscript.

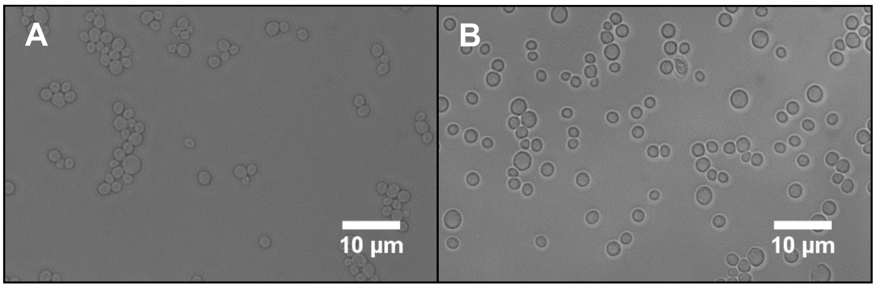

FIG S2 Morphology of *P. pastoris* during retentostat cultivation. Representative bright field micrographs of cells grown for 1 (A) and 25 days (B) in retentostat culture.

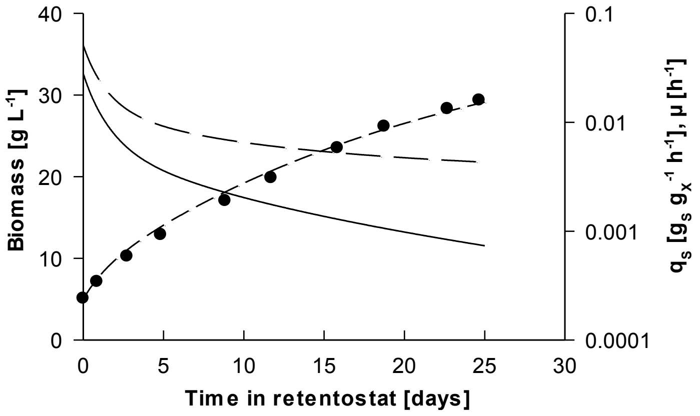

FIG S3 Example of non-linear regression analysis of viable biomass for one retentostat. Viable biomass (closed circles), non-linear regression of viable biomass (short dashed line) and resulting glucose uptake rate (q_S, long dashed line) as well as specific growth rate (μ, solid line).
